# Chaperone-mediated autophagy dysregulation during aging impairs hepatic fatty acid oxidation via accumulation of NCoR1

**DOI:** 10.1016/j.molmet.2023.101784

**Published:** 2023-07-29

**Authors:** You-Jin Choi, Sung Ho Yun, Jihyeon Yu, Yewon Mun, Wonseok Lee, Cheon Jun Park, Byung Woo Han, Byung-Hoon Lee

**Affiliations:** 1 College of Pharmacy and Research Institute of Pharmaceutical Sciences, Seoul National University, Seoul 08826, Republic of Korea; 2 Division of Life Science, Korea Polar Research Institute, Incheon 21990, Republic of Korea

**Keywords:** Aging, Chaperone-mediated autophagy, PPARα, NCoR1, Fatty acid oxidation

## Abstract

**Objective:**

Alterations in lipid metabolism are associated with aging and age-related diseases. Chaperone-mediated autophagy (CMA) is a lysosome-dependent process involved in specific protein degradation. Heat shock cognate 71 kDa protein (Hsc70) recognizes cytosolic proteins with KFERQ motif and allows them to enter the lysosome via lysosome-associated membrane glycoprotein 2 isoform A (LAMP2A). CMA deficiency is associated with dysregulated lipid metabolism in the liver. In this study, we examined the effect of CMA on lipid metabolism in the aged liver.

**Methods:**

12-week-old and 88-week-old mice were employed to assess the effect of aging on hepatic CMA activity. We generated CMA-deficient mouse primary hepatocytes using siRNA for Lamp2a and liver-specific LAMP2A knockdown mice via adeno-associated viruses expressing short hairpin RNAs to investigate the influence of CMA on lipid metabolism.

**Results:**

We noted aging-induced progression toward fatty liver and a decrease in LAMP2A levels in total protein and lysosomes. The expression of genes associated with fatty acid oxidation was markedly downregulated in the aged liver, as verified in CMA-deficient mouse primary hepatocytes. In addition, the aged liver accumulated nuclear receptor corepressor 1 (NCoR1), a negative regulator of peroxisome proliferator-activated receptor α (PPARα). We found that Hsc70 binds to NCoR1 via the KFERQ motif. Lamp2a siRNA treatment accumulated NCoR1 and decreased the fatty acid oxidation rate. Pharmacological activation of CMA by AR7 treatment increased LAMP2A expression, leading to NCoR1 degradation. A liver-specific LAMP2A knockdown via adeno-associated viruses expressing short hairpin RNAs caused NCoR1 accumulation, inactivated PPARα, downregulated the expression of fatty acid oxidation-related genes and significantly increased liver triglyceride levels.

**Conclusions:**

Our results elucidated a novel PPARα regulatory mechanism involving CMA-mediated NCoR1 degradation during aging. These findings demonstrate that CMA dysregulation is crucial for the progression of aging-related fatty liver diseases.

## Highlights

• Aging decreases LAMP2A levels and increases CMA substrate levels in liver.
• Aging inhibits fatty acid oxidation via NCoR1/PPARα pathway.
• CMA regulates PPARα through NCoR1 degradation.

## 1 Introduction

Aging is accompanied by chronic inflammation and dysregulated lipid metabolism, which may contribute to metabolic syndrome. The prevalence of metabolic syndrome is 7% among those aged 20–29 but increases to 44% among those aged 60–69 [1]. Non-alcoholic fatty liver disease (NAFLD) is used to characterize obese and non-drinking patients with steatotic liver disease [2]. NAFLD is a liver manifestation of metabolic syndrome that is associated with obesity, insulin resistance, hypertension, and dyslipidemia. NAFLD can result in liver fibrosis, cirrhosis, and hepatocellular carcinoma due to the inflammation and liver damage. Although the underlying mechanisms are not fully understood, aging is widely believed to increase susceptibility to NAFLD [3]. This may be due to the increased prevalence of metabolic syndrome, such as obesity and diabetes, or metabolic changes that occur during aging. Aging is considered to promote NAFLD through various mechanisms, including dysregulated nutrient sensing, impaired autophagy, and mitochondrial dysfunction [4].

Previous studies have shown that fatty acid oxidation and the expression of many related genes decline with aging [5,6]. Peroxisome proliferator-activated receptor α (PPARα) regulates genes involved in lipid metabolism. PPARα regulates fatty acid oxidation along with retinoid X receptor (RXR) as a heterodimer, which binds peroxisome proliferator response element (PPRE). Expression of carnitine palmitoyl transferase 1α (Cpt1α), which initiates the mitochondrial oxidation of long-chain fatty acids, is downregulated during aging due to reduced PPARα transcription [6]. PPARα inactivation is linked to the upregulation of advanced glycation end product receptor (RAGE) in the aged liver, and RAGE suppression improves fatty liver by PPARα activation and increased fatty acid oxidation [7]. Furthermore, hepatocyte-specific PPARα deletion disrupts fatty acid oxidation in mice, resulting in progression toward fatty liver even on a standard diet during aging [5]. These studies suggest that PPARα plays an essential role in aging-related fatty liver. However, it is unclear how aging affects PPARα mRNA and protein levels.

Autophagy is a self-degradative process that is crucial for regulating energy levels in response to nutrient stress. Macroautophagy, microautophagy, and chaperone-mediated autophagy (CMA), which are defined types of autophagy, facilitate lysosomal degradation of cytosolic components. CMA is a selective, lysosomal-dependent protein degradation pathway. Cytosolic proteins with a KFERQ-like motif can be recognized by Heat shock cognate 71 kDa protein (Hsc70) and move into the lysosome through lysosome-associated membrane glycoprotein 2 isoform A (LAMP2A). CMA plays an essential role in physiological processes, such as proteostasis, glucose and lipid metabolism, and immune responses. Recent data have emphasized the significance of CMA in lipid metabolism. Experimental and clinical studies have demonstrated that inhibition of CMA-induced perilipin (PLIN) degradation reduces lipid droplet breakdown [8,9]. Hsc70 binds to the KFERQ motif of PLIN, and AMP-activated protein kinase (AMPK) phosphorylates PLIN, allowing it to detach from lipid droplets and be degraded by CMA [10]. Moreover, a study with CMA-defective mouse models showed that tricarboxylic acid (TCA) cycle-related enzymes are regulated by CMA under starvation conditions, implicating a role for CMA in glucose and lipid metabolism [11].

The functional decline of CMA has been observed in aged rodent tissues and cells derived from aged individuals [[12], [13], [14], [15]]. Changes in the lipid components of the lysosomal membrane reduce the stability of LAMP2A, and LAMP2A protein levels in the mouse liver decrease with age [14]. Liver-specific LAMP2A-deficient mice develop hepatic steatosis at an early age, suggesting that CMA may play a role in aging-related NAFLD progression [16]. This was confirmed in aged mice expressing an inducible hepatocyte-specific LAMP2A transgene, which showed lower hepatic lipid levels and improved liver function [15].

Despite evidence of dysregulation of CMA in the aged liver, the underlying molecular mechanism has not been established. To identify the molecular players responsible for the alteration in lipid metabolism during aging, we examined gene expression profiles in aged mouse livers and CMA-deficient primary hepatocytes. We report the inactivation of PPARα target genes, as well as the accumulation of nuclear receptor corepressor 1 (NCoR1; a negative regulator of PPARα) in the aged liver. Here, we describe how CMA-dependent NCoR1 degradation regulates PPARα during aging and present *in vivo* evidence of the pathological significance of CMA-dependent changes in lipid metabolism.

## 2 Materials and methods

### 2.1 Animal studies

Male C57BL/6 mice were obtained from Orient Bio (Seongnam, Korea). To investigate the changes in CMA and lipid metabolism during aging, 3- and 22-month-old mice were used (n = 7). The adeno-associated virus 8 encoding Lamp2a-specific short hairpin RNA (AAV8-shLamp2a; generated by VectorBuilder) was intraperitoneally injected with 10^11^ viral particles into male 8-week-old mice (n = 4). The Institutional Animal Care and Use Committee of Seoul National University approved all animal studies.

### 2.2 Serum biochemistry and quantification of triglyceride and cholesterol

Whole blood samples were collected and separated by centrifugation. The levels of alanine aminotransferase (ALT), aspartate aminotransferase (AST), triglycerides, and cholesterol in serum were examined by an Automated Chemistry Analyzer (Prestige 24I; Tokyo Boeki Medical System). Lipids were extracted from liver tissues using chloroform/methanol (2:1, v/v) and hepatic triglycerides and cholesterol levels were determined using colorimetric assay kits (TR0100, MAK043; Sigma-Aldrich). β-Hydroxybutyrate levels were measured using a β-hydroxybutyrate assay kit (MAK041; Sigma-Aldrich) according to the manufacturer's protocol.

### 2.3 Liver histology

The liver tissues were fixed in 10% neutral buffered formalin, embedded in paraffin, and stained with hematoxylin and eosin (H&E). For NCoR1 immunostaining, liver paraffin sections were incubated with NCoR1 antibody (1:200 dilution, PA1-844A, Thermo Fisher Scientific) and counterstained with hematoxylin. Liver frozen sections were stained with Oil-Red O to visualize lipid droplets.

### 2.4 Isolation and treatment of mouse primary hepatocytes

Primary hepatocytes were isolated from male C57BL/6 mice aged 8–10 weeks. The isolation procedure involved two-step perfusion with calcium and magnesium-free Hanks' salt solution followed by a William's E medium containing collagenase type IV (C5138, Sigma-Aldrich). The isolated hepatocytes were plated in collagen-coated dishes (CB40236, Thermo Fisher) using William's E medium (W4125, Sigma-Aldrich) supplemented with 10% fetal bovine serum (FBS), 100 nM dexamethasone, and 100 nM insulin. After allowing 4 h for adhesion, the cells were washed with PBS and the medium was replaced with William's E medium containing 10% FBS prior to the specified treatments. Cells were incubated at 37 °C with air containing 5% CO_2_. Chemicals used in the study, including AR7 (SML0921), cycloheximide (01810), and leupeptin (L2884) were purchased from Sigma-Aldrich. For silencing Lamp2a expression, siRNA for Lamp2a (siLamp2a) obtained from Bioneer (#16784-2) was transiently transfected into the cells at a concentration of 60 pmol using RNAiMax (13778-075, Invitrogen) for a duration of 72 h. Western blot analysis and PCR determined transfection efficiency.

### 2.5 Western blot analysis

Cell pellets and liver tissue were lysed in modified RIPA buffer (50 mM Tris–HCl pH 8.0, 150 mM NaCl, 1% NP-40, 1% sodium deoxycholate) and centrifuged at 14,000 rpm for 15 min. Supernatants were collected, electrophoresed on 6–15% SDS-PAGE gel, and transferred onto the PVDF membrane. Western blots were probed with the following antibodies: NCoR1 (#5948), PKM2 (#4053) from Cell Signaling Technology; β-actin (sc-47778), α-tubulin (sc-5286), PPARα (sc-398394), and Lamin B (sc-374015) from Santa Cruz Biotechnology; LAMP2A (ab18528) from Abcam; PLIN2 (NB110-40877) and Hsc70 (NB120-2788) from Novus Biologicals; LAMP1 (1d4b) from DSHB.

### 2.6 Isolation of lysosomes, co-immunoprecipitation, and nuclear fractionation

Lysosome isolation was performed with 100 mg of liver tissue using a lysosome enrichment kit (89839, Thermo Fisher Scientific). Co-immunoprecipitation was performed using a specific antibody against Hsc70 overnight and subjected to bind protein G agarose bead. The immunoprecipitated bead was washed with modified RIPA buffer three times and boiled with SDS sample buffer. After centrifugation, the lysates were subjected to western blotting. For nuclear fractionation, cells were lysed in mitochondria extraction buffer (20 mM HEPES, pH 7.5, 1.5 mM MgCl_2_, 10 mM KCl, 1 mM DTT, 1 mM EDTA, 1 mM EGTA, 0.1 mM PMSF, 250 mM Sucrose). The lysates were incubated on ice for 1 h, followed by centrifugation at 1000 *g* at 4 °C for 4 min. The supernatant was considered cytosol fraction and further cleared by centrifugation at 15,000 rpm at 4 °C for 20 min. The pellets were washed twice and then incubated with modified RIPA buffer for 30 min. After centrifugation at 15,000 rpm at 4 °C for 20 min, the supernatant was considered nuclear fraction.

### 2.7 Quantitative real-time polymerase chain reaction (qRT-PCR)

Total RNA was extracted by Easy-Blue™ Toal RNA extraction kit (17061, Intron Biotechnology). The cDNA was synthesized using QuantiTect Reverse Transcription Kit (205313, Qiagen). qRT-PCR was conducted on a Stepone™ Real-time PCR system (Applied Biosystems) using iTaq™ Universal SYBR Green Supermix kit (1725121, Bio-rad) following the manufacturer's instruction. The gene-specific primers are presented in Table 1.

**Table 1:** Gene-specific primers used in qRT-PCR.

Gene symbol	Accession no.	Primer	Sequence
*Acaca*	NM_133360.3	Forward	5′ AGCAGATCCGCAGCTTG 3′
		Reverse	5′ ACCTCTGCTCGCTGAGTGC 3′
*Acox1*	NM_001377521.1	Forward	5′ GCCATTCGATACAGTGCTGTGAG 3′
		Reverse	5′ CCGAGAAAGTGGAAGGCATAGG 3′
*Cd36*	XM_006535620.3	Forward	5′ CCATTCCTCAGTTTGGTTCC 3′
		Reverse	5′ TGCATTTGCCAATGTCTAGC 3′
*Cpt1α*	NM_013495.2	Forward	5′ GGCATAAACGCAGAGCATTCCTG 3′
		Reverse	5′ CAGTGTCCATCCTCTGAGTAGC 3′
*Cpt2*	NM_009949.2	Forward	5′ GATGGCTGAGTGCTCCAAATACC 3′
		Reverse	5′ GCTGCCAGATACCGTAGAGCAA 3′
*Dgat2*	NM_026384.3	Forward	5′ CTGGCTGGCATTTGACT 3′
		Reverse	5′ TCTATGGTGTCTCGGTTGA 3′
*Fasn*	NM_007988.3	Forward	5′ TTCCGTCACTTCCAGTTAGAG 3′
		Reverse	5′ TTCAGTGAGGCGTAGTAGACA 3′
*Lamp2a*	NM_001017959.2	Forward	5′ AGGTGCTTTCTGTGTCTAGAGCGT 3′
		Reverse	5′ AGAATAAGTAGTACTCCTCCCAGAGCTGC 3′
*Acadl*	NM_007381.4	Forward	5′ CCGATGTTCTCATTCTGG 3′
		Reverse	5′ TGGCGTTCGTTCTTACTC 3′
*Mttp*	NM_001163457.2	Forward	5′ GTGGAGGAATCCTGATGGTGA 3′
		Reverse	5′ TGATCTTAGGTGTACTTTTGCCC 3′
*Ncor1*	NM_001252313.1	Forward	5′ TTCTGAAATTATTGATGGTCTTTCTG 3′
		Reverse	5′ ACAGAAAGCTGACGCATTTG 3′
*Pkm2*	XM_011242674.2	Forward	5′ GTCTGGAGAAACAGCCAAGG 3′
		Reverse	5′ CGGAGTTCCTCGAATAGCTG 3′
*Plin2*	NM_001403711.1	Forward	5′ TCTACTCCGTATTCCGCAATGC 3′
		Reverse	5′ TTCGGTCCAGACAGACGTTT 3′
*Pparα*	NM_001113418.1	Forward	5′ GACCTGAAAGATTCGGAAACT 3′
		Reverse	5′ CTCTTCTCGGCCATACAC 3′
*Srebf1c*	XM_006532716.4	Forward	5′ TGGAGACATCGCAAACAAG 3′
		Reverse	5′ GGTAGACAACAGCCGCATC 3′
*Actb*	NM_007393.5	Forward	5′ CATTGCTGACAGGATGCAGAAGG 3′
		Reverse	5′ TGCTGGAAGGTGGACAGTGAGG 3′

### 2.8 Measurement of fatty acid oxidation rate

Fatty acid oxidation rate was measured on a Seahorse XFe96 analyzer using the Seahorse XF palmitate oxidation stress test kit (103693-100, Agilent Technologies). Briefly, primary hepatocytes were plated in 96-well Seahorse culture plates and transfected with siLamp2a. After two days, the culture medium was replaced with a substrate-limited growth medium containing 1% FBS, 0.5 mM glucose, 1 mM glutamine, 0.5 mM l-carnitine. The following day, the cells were switched to a substrate-limited assay medium supplemented with BSA or palmitate-BSA (167 μM). The oxygen consumption rate (OCR) was measured by sequentially injecting 1.5 μM oligomycin, 1 μM FCCP, and 0.5 μM antimycin A/rotenone.

### 2.9 Nile red staining

Mouse primary hepatocytes were plated in 96-well plates with clear bottoms and black sides at a density of 1 × 10^4^ cells per well. The cells were transfected with siLamp2a and incubated for 48 h. Following the transfection, the cells were treated with 100 μM oleic acid in a serum-free medium containing 1% fatty acid-free BSA for 24 h. After the treatment period, the cells were fixed using 4% paraformaldehyde and stained with 5 μg/mL Hoechst 33258 (94403, Sigma-Aldrich) and 0.5 μg/mL Nile red solution (19123, Sigma-Aldrich). Nile red fluorescence was measured using excitation/emission wavelengths of 488/550 nm, and the measurements were normalized by Hoechst 33258 fluorescence at 341/452 nm using the Cytation 3 system (BioTek).

### 2.10 Confocal microscopy

Primary hepatocytes were transfected with Lamp2a-mCherry plasmid using lipofectamine 2000 (11668, Thermo Fisher Scientific) and then treated with leupeptin for 24 h. Cells were fixed with 4% paraformaldehyde and permeabilized by Triton X-100. Fixed samples were incubated with NCoR1 antibody (1:250, PA1-844A, Thermo Fisher Scientific) at 4 °C for overnight, followed by sequential incubation with Alexa Fluor® 488 dye-labeled secondary antibody. The specimens were mounted using ProLong™ Diamond Antifade Mountant with DAPI (P36962, Thermo Fisher Scientific) and analyzed with a TCS SP8 confocal laser scanning microscope (Leica Microsystems).

### 2.11 Molecular docking and binding energy calculation

To stabilize helix structures of the NCoR1 KFERQ-like peptides (Hsc70-binding peptides) for molecular docking simulation, two more amino acid residues were included before and after the NCoR1 KFERQ-like peptides; NCoR1 ^346^FPEIRKQRE^354^, ^448^FKDKFIQHP^456^, ^1326^PKQIKRESP^1334^, and ^2222^KKQEIFRKL^2230^. Additionally, Ala mutations were introduced to significant binding residues of the NCoR1 KFERQ-like peptides for comparative analysis of the docking results. Resultant sequences of the mutant NCoR1 KFERQ-like peptides are NCoR1 ^346^FPAARKARE^354^, ^448^FKAKFAAHP^456^, ^1326^PKAAKRASP^1334^, and ^2222^KKAAAFRKL^2230^. Structure files for the wildtype NCoR1 KFERQ-like peptides and the mutants were created using ColabFold v1.5.2-patch: AlphaFold2 with MMseqs2 [17].

Since amino acid sequences of Hsc70 substrate binding domain (SBD) from rat and human are identical, the rat Hsc70 SBD structure (PDB code: 7HSC) was employed as the input protein model for molecular docking simulations of human NCoR1 KFERQ-like peptides. The HADDOCK 2.4 webserver was used for the protein-peptide docking processes, resulting in binding modes of the NCoR1 KFERQ-like peptides to Hsc70 SBD [18]. The default setting for sampling and clustering was used for the docking. The number of structures for rigid-body docking was 1000, and number of structures for semi-flexible refinement and number of structures for final refinement was 200 each. RMSD clustering method was used with 5 cut-off and minimum cluster size was 4.0.

For binding energy calculation, each of Hsc70 and the KFERQ-like peptides was prepared using Preparation Wizard in Schrödinger suite [19]. Binding free energy (ΔG_bind_) of NCoR1 KFERQ-like peptides to Hsc70 was calculated using Molecular Mechanics/Generalized Born Surface Area (MM/GBSA) method. The solvation model of VSGB (variable-dielectric generalized born model) with OPLS2004 force field [20] was applied, and the binding free energy calculation was performed using Prime [21,22] MM/GBSA module in Schrödinger suite [19].

### 2.12 Statistical analysis

Statistical analysis was performed using GraphPad Prism 7.0 (GraphPad Software, Inc., CA, USA). Data were presented as mean with standard deviation (SD) and considered statistically significant at p < 0.05. When only two groups were compared, a two-tailed Student's t-test was used. Multiple comparisons were carried out using one-way analysis of variance (ANOVA) followed by Tukey's multiple comparison test.

## 3 Results

### 3.1 Aging decreases LAMP2A levels and increases CMA substrate levels in liver

**Figure 1:**
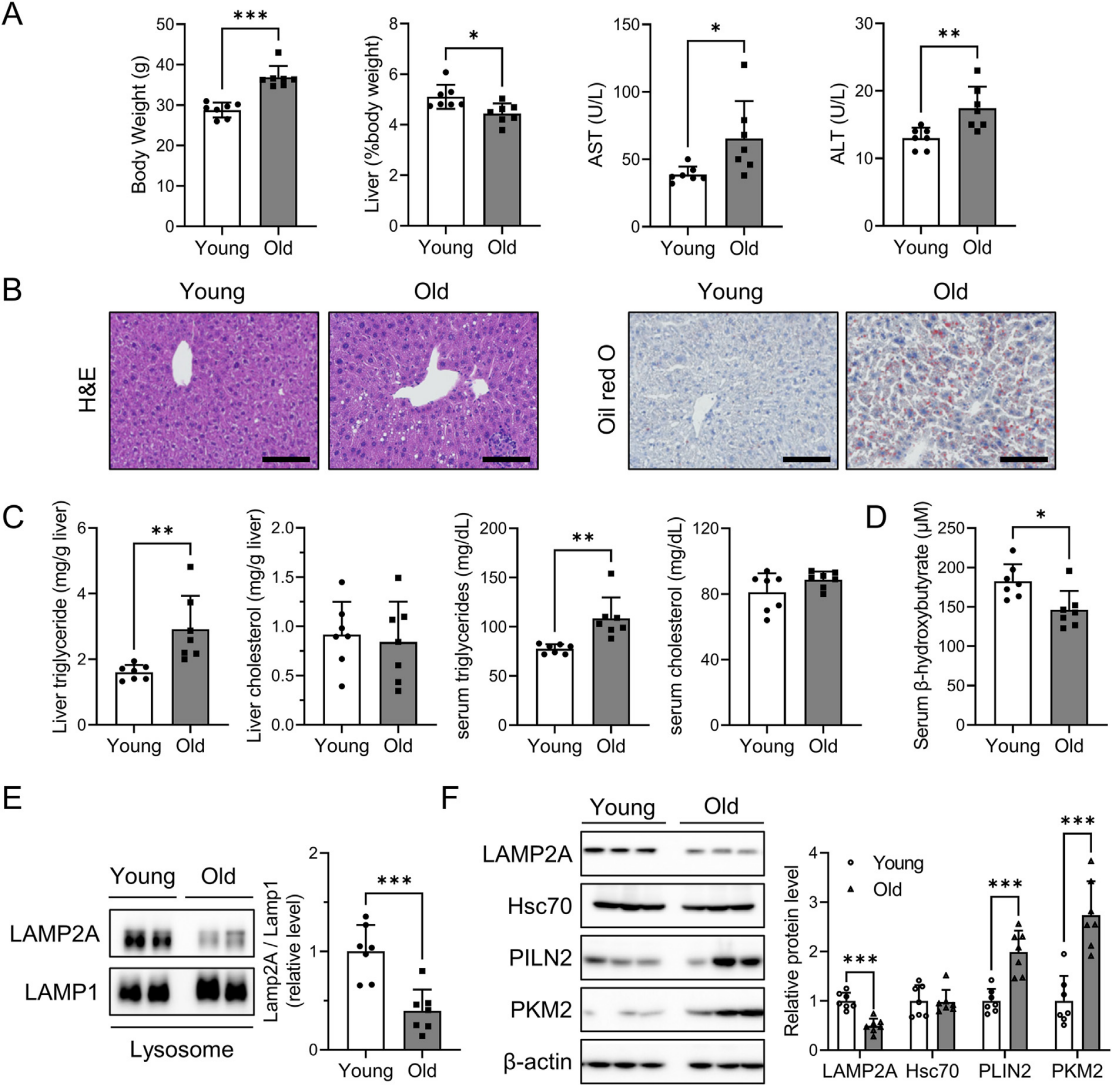
**Increased lipid accumulation and CMA inhibition in the aged liver**, Livers from mice of different ages (3 and 22 months) were used to examine the effect of aging on hepatic changes. (A) Body and liver weights were measured, and serum AST and ALT levels were determined as described in the Materials and methods section. (B) Representative images of H&E and oil red O staining shows the livers of young and old mice, with a scale bar of 100 μm. (C) Triglyceride and cholesterol levels in liver and serum were quantified as described in the Materials and methods section. (D) β-Hydroxybutyrate levels were measured in serum samples obtained from young and old mice. (E) Lysosomal LAMP2A protein expression was examined using western blotting. LAMP1 was used as a lysosomal marker and loading control. (F) Hepatic protein levels of LAMP2A, Hsc70 and CMA substrates were quantified using western blotting. qRT-PCR was performed to measure hepatic mRNA levels of LAMP2A and CMA substrates. Each bar represents the mean ± SD (n = 7). Asterisks above the bars indicate significant differences compared to young mice using Student's t-test: ∗p < 0.05; ∗∗p < 0.01; ∗∗∗p < 0.001.

Previous studies have shown that CMA activity declines with age, which can be attributed to the decreased stability of LAMP2A in the lysosomal membrane [14,16]. We first examined whether aging could cause lipid accumulation through CMA inhibition in the liver. We compared the liver histology and function in young and old male mice. The old mice showed significantly higher AST and ALT levels (Figure 1A). As shown in Figure 1B, cellular vacuolization was observed in the aged livers. Liver triglycerides also increased by 1.5-fold in old mice (Figure 1C), as confirmed by oil red O staining (Figure 1B). Consistent with these results, serum triglyceride levels were significantly increased in old mice (Figure 1C). Additionally, we observed a decrease in serum β-hydroxybutyrate levels in the aged mice, indicating reduced fatty acid oxidation (Figure 1D).

The CMA activity was also evaluated in the livers of young and old mice. Because CMA activity is directly related to the amount of LAMP2A, we examined the levels of LAMP2A using western blot analysis [11]. LAMP2A levels in total protein and lysosomes were lower in the aged liver, but mRNA levels remained unchanged (Figure 1E, F, Suppl 1). Moreover, the protein levels of Hsc70, a key component in CMA, were unaffected by aging (Figure 1F). Levels of CMA substrate proteins, such as PLIN2 and PKM2, were significantly elevated in the aged liver, which was not regulated by gene transcription (Figure 1F). These findings showed that the aged liver had lower levels of LAMP2A, indicating CMA inhibition and higher levels of triglycerides.

### 3.2 Aging inhibits fatty acid oxidation via NCoR1/PPARα pathway

**Figure 2:**
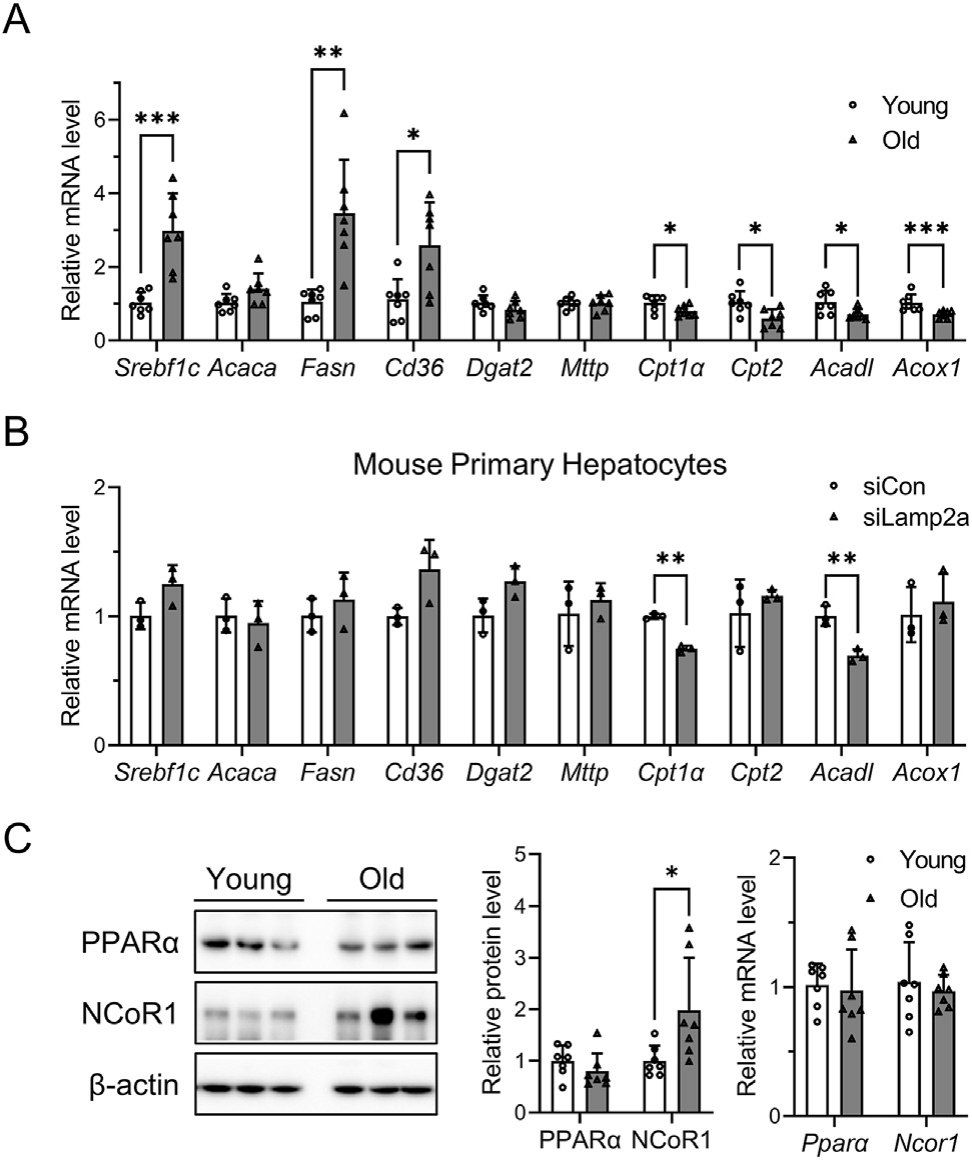
**Inactivation of PPARα by NCoR1 accumulation in CMA-deficient hepatocytes**. Livers from mice of different ages (3 and 22 months) were used to examine the effect of aging on hepatic changes. Primary hepatocytes were isolated from 8- to 10-week-old male C57BL/6 mice and transfected with either siCon or siLamp2a for 72 h. (A, B) Quantification of lipid metabolism-related genes was performed using qRT-PCR. (C) Western blotting was conducted to measure hepatic protein levels of NCoR1 and PPARα. Hepatic mRNA levels of NCoR1 and PPARα were determined using qRT-PCR. The *in vivo* data are presented as mean ± SD (n = 7), while the *in vitro* data are shown as the mean ± SD (n = 3). Asterisks above the bars indicate significant differences compared to young mice or control using Student's t-test: ∗p < 0.05; ∗∗p < 0.01; ∗∗∗p < 0.001.

To better understand the underlying molecular mechanism by which CMA inhibition causes aging-related fatty liver, we first examined the gene expression profiles of aged mouse livers using the NCBI Gene Expression Omnibus (GEO) database (GSE3150) [23]. When we compared an array of hepatic lipid metabolism genes in young and old mice (4 and 22 months), we found that the expression of genes responsible for fatty acid oxidation was downregulated (*Cpt1α*, *Cpt2*, *Acadl*, *Acox1*; Suppl 2). The expression levels of genes associated with lipid metabolism were validated using an animal model. qRT-PCR analysis showed that the expression of fatty acid oxidation-related genes was significantly downregulated in aged livers (Figure 2A). We also confirmed that the levels of known targets of PPARα, including *Cpt1α* and *Acadl*, decreased in mouse primary hepatocytes transfected with siLamp2a (Figure 2B). As PPARα enhances fatty acid oxidation, its inactivation is considered to play an important role in the development of NAFLD [24]. Thus, we investigated the mRNA and protein expression of PPARα in the aged livers. However, PPARα mRNA and protein levels were unchanged in aged livers (Figure 2C). Remarkably, NCoR1, a negative regulator of PPARα, accumulated in the aged liver and was not accompanied by an increase in transcription of NCoR1 (Figure 2C). These results led us to hypothesize that CMA dysregulation may result in NCoR1 accumulation with diminished fatty acid oxidation.

### 3.3 NCoR1 is a substrate of CMA

**Figure 3:**
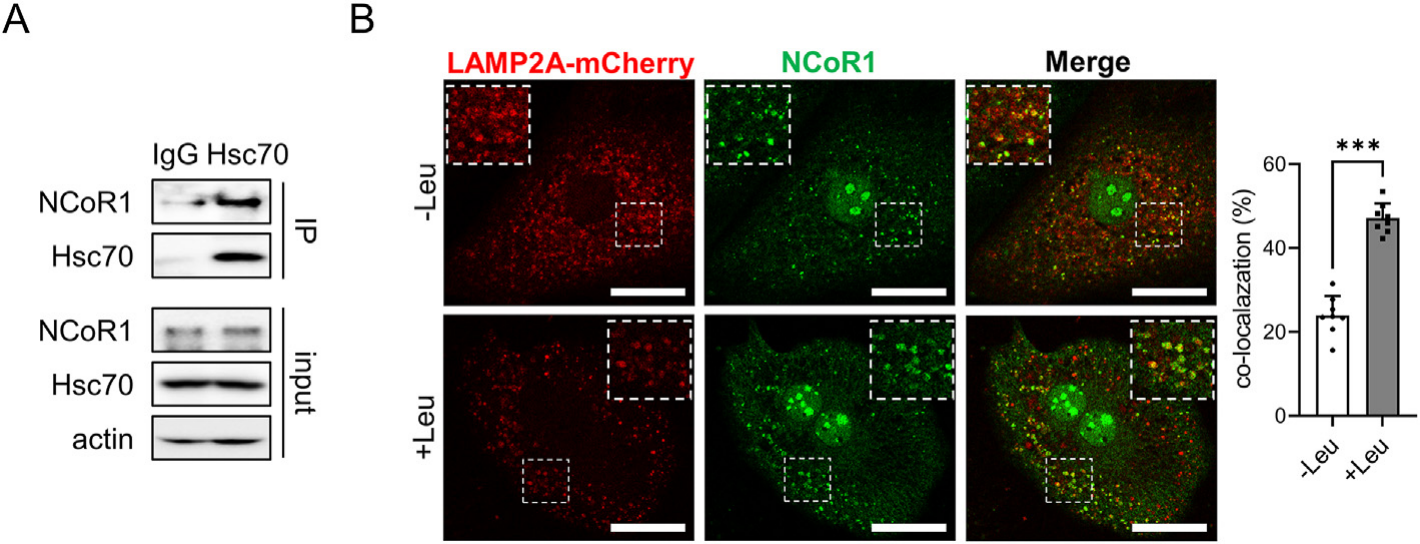
**The direct association between NCoR1 and Hsc70**. Primary hepatocytes were isolated from 8- to 10-week-old male C57BL/6 mice and treated with siRNA or chemicals as indicated. (A) Co-immunoprecipitation of endogenous Hsc70 and NCoR1 was performed with IgG or Hsc70 antibody in primary hepatocytes. (B) Mouse primary hepatocytes expressing LAMP2A-mCherry (red) were incubated with leupeptin 100 μM for 24 h. The fixed cells were subjected to immunofluorescence analysis with antibodies against NCoR1 (green). Nuclei were stained with DAPI (blue). Representative confocal microscopy images are shown, and the scale bar is set to 10 μm. The percentage of LAMP2A-mCherry spots colocalizing with NCoR1 was plotted in left panel. Each bar represents the mean ± SD. Asterisks above the bars indicate significant differences compared to the control using Student's t-test: ∗∗∗p < 0.001.

Most CMA substrates contain a KFERQ-like motif for lysosomal targeting by Hsc70 [25]. Previous study found that NCoR1 contains a KFERQ-like motif and that CMA contributes to the elimination of misfolded NCoR1 in lung cancer cells [26]. While previous research has focused on the effects of CMA on misfolded NCoR1 degradation and oncogenic growth, we wanted to examine NCoR1 regulation by CMA in terms of lipid metabolism. To determine whether NCoR1 is a CMA substrate, the NCoR1 peptide sequence was analyzed using KFERQ finder software v0.8 [27]. The software revealed the presence of four canonical KFERQ-like motifs in human and mouse NCoR1 (Table 2). A co-immunoprecipitation assay was performed to evaluate the direct association between NCoR1 and Hsc70. The interaction between NCoR1 and Hsc70 was confirmed via western blotting (Figure 3A). Additionally, we observed co-localization of LAMP2A and NCoR1, which exhibited an increase when cells were treated with leupeptin (Figure 3B).

**Table 2:** KFERQ-like motif in NCoR1.

Protein	Residues	Motif sequence
hNCoR1	348–352	EIRKQ
	450–454	DKFIQ
	1317–1321	QIKRE
	2210–2214	QEIFR
mNCoR1	348–352	EIRKQ
	450–454	DKFIQ
	1328–1332	QIKRE
	2224–2228	QEIFR

**Figure 4:**
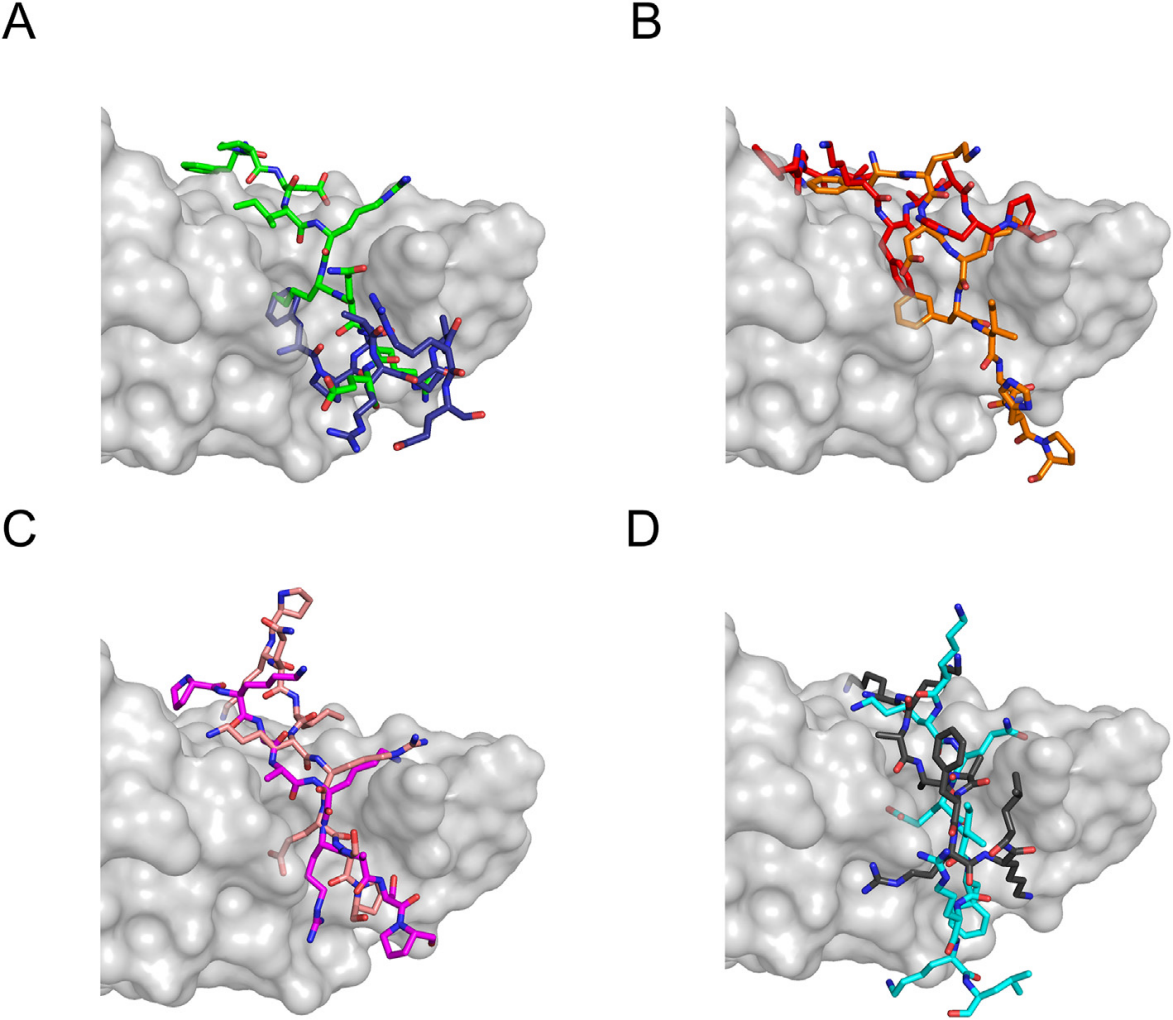
**Molecular docking results of the NCoR1 KFERQ-like peptides into Hsc70 SBD**. NCoR1 KFERQ-like peptides are bound to Hsc70 SBD and the binding modes of the wildtype are compared to those of their mutants; (A) NCoR1 ^346^FPEIRKQRE^354^ (green) and ^346^FPAARKARE^354^ (deepblue), (B) NCoR1 ^448^FKDKFIQHP^456^ (orange) and ^448^FKAKFAAHP^456^ (red), (C) NCoR1 ^1326^PKQIKRESP^1334^ (salmon) and ^1326^PKAAKRASP^1334^ (magenta), and (D) ^2222^KKQEIFRKL^2230^ (cyan) and ^2222^KKAAAFRKL^2230^ (grey30). Hsc70 SBD is shown as surface view in grey80 and the KFERQ-like peptides are shown as stick model. The aforementioned colors of KFERQ-like peptides correspond to the representation of carbon atoms, while nitrogen and oxygen atoms are depicted in blue and red, respectively.

To provide further evidence of the interaction between Hsc70 and the NCoR1 KFERQ-like motif, we conducted molecular docking simulations and binding energy calculations. Since Hsc70 and Heat-shock protein 70 kDa (Hsp70) share 91% identical amino acid sequences in the SBD and play similar cellular roles [28], we used the structure of Hsp70 SBD in complex with the KFERQ-like motif (PDB code: 4PO2) as a reference model for docking simulations of the NCoR1 KFERQ-like motifs to Hsc70 SBD. Control docking was conducted on PDB 4PO2 using the HADDOCK 2.4 webserver [18]; the HADDDOCK score was 115.4. After validating the reliability of the docking tool, we conducted molecular docking simulations of the NCoR1 KFERQ-like motifs to Hsc70 SBD. While all KFERQ-like residues of the Wildtype NCOR1 peptides ^346^FPEIRKQRE^354^, ^448^FKDKFIQHP^456^, ^1326^PKQIKRESP^1334^, and ^2222^KKQEIFRKL^2230^ showed interaction with peptide-binding pocket at β subdomain of Hsc70 SBD, the Ala mutated peptides only partially created interaction with Hsc70 SBD (Figure 4).

The HADDOCK scores between Hsc70 SBD and the wildtype NCoR1 peptides, ^346^FPEIRKQRE^354^, ^448^FKDKFIQHP^456^, ^1326^PKQIKRESP^1334^, and ^2222^KKQEIFRKL^2230^, were −86.8, −97.8, −81.5, and −88.2, respectively (Table 3). The corresponding HADDOCK scores for the NCoR1 mutants, ^346^FPAARKARE^354^, ^448^FKAKFAAHP^456^, ^1326^PKAAKRASP^1334^, and ^2222^KKAAAFRKL^2230^, were −67.2, −82.5, −64.9, and −73.9, respectively (Table 3). The increased values of the HADDOCK scores, 19.6, 15.3, 16.6, and 14.3, respectively, indicate that mutations in the NCoR1 KFERQ-like peptides destabilize the interaction between Hsc70 SBD and NCoR1. Moreover, we conducted a comprehensive analysis for binding energy calculation using the MM/GBSA method. MM/GBSA combines molecular mechanics force, which analyzes interactions between atoms with a generalized Born model that estimates the solvation effects. Table 4 shows the ΔG_bind_ of the Wildtype and Ala mutated NCoR1 KFERQ-like peptides to Hsc70 SBD. The increased values of ΔG_bind_ were 21.3, 11.7, 16.1, and 9.9 kcal/mol, respectively, providing additional evidence of a destabilizing interaction between Hsc70 SBD and NCoR1 due to mutations in the NCoR1 KFERQ-like peptides. The HADDOCK score and MM/GBSA method have different calculation approaches in molecular docking simulation. According to our result, both the HADDOCK score and the MM/GBSA value not only prove the interaction between Hsc70 SBD and NCoR1 KFERQ-like peptides, but also highlight the significance of KFERQ-like residues of NCoR1 for binding to Hsc70 SBD. These results indicate that NCoR1 can be delivered to the lysosomal membrane by Hsc70.

**Table 3:** HADDOCK scores from docking result of Wildtype and Ala-mutated NCoR1 KFERQ-like peptide to Hsc70 SBD, HADDOCK score is determined by combining van der Waals intermolecular energy, electrostatic intermolecular energy, desolvation energy, and distance restraints energy. More negative value of HADDOCK score indicates higher binding affinity of peptides to protein. Ala-mutated residue is underlined and bolded.

	HADDOCK score	Change in HADDOCK score
NCoR1 ^346^FPEIRKQRE^354^	−86.8 ± 4.5	19.6
NCoR1 ^346^FP**AA**RK**A**RE^354^	−67.2 ± 5.6	
NCoR1 ^448^FKDKFIQHP^456^	−97.8 ± 8.8	15.2
NCoR1 ^448^FK**A**KF**AA**HP^456^	−82.5 ± 2.8	
NCoR1 ^1326^PKQIKRESP^1334^	−81.5 ± 4.1	16.6
NCoR1 ^1326^PK**AA**KR**A**SP^1334^	−64.9 ± 8.6	
NCoR1 ^2222^KKQEIFRKL^2230^	−88.2 ± 4.9	14.3
NCoR1 ^2222^KK**AAA**FRKL^2230^	−73.9 ± 2.3

**Table 4:** Binding free energy (ΔG_bind_) of Wildtype and Ala-mutated NCoR1 KFERQ-like peptide bound to Hsc70 SBD is calculated using MM/GBSA method, MM/GBSA estimates the binding free energy of protein-peptide complexes by considering both the molecular interactions and the solvation effects. More negative value of binding free energy indicates higher binding affinity of peptides to protein. Ala-mutated residue is underlined and bolded.

	ΔG_bind_ (kcal/mol)	ΔG_bind_ Mutant − ΔG_bind_ Wildtype (kcal/mol)
NCoR1 ^346^FPEIRKQRE^354^	−80.6	21.3
NCoR1 ^346^FP**AA**RK**A**RE^354^	−59.3	
NCoR1 ^448^FKDKFIQHP^456^	−85.3	11.7
NCoR1 ^448^FK**A**KF**AA**HP^456^	−73.6	
NCoR1 ^1326^PKQIKRESP^1334^	−80.4	16.1
NCoR1 ^1326^PK**AA**KR**A**SP^1334^	−64.3	
NCoR1 ^2222^KKQEIFRKL^2230^	−76.5	9.9
NCoR1 ^2222^KK**AAA**FRKL^2230^	−66.6	

**Figure 5:**
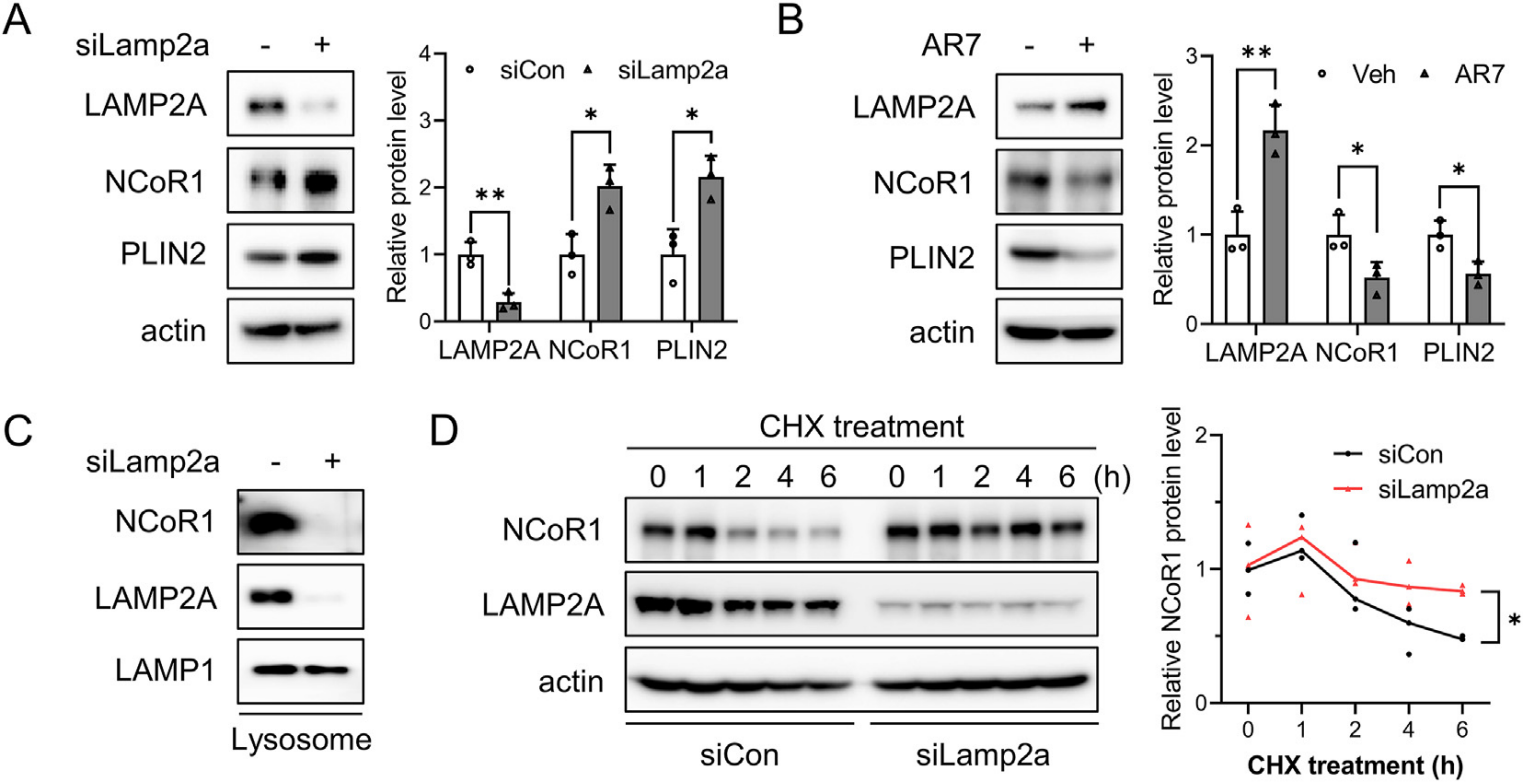
**Selective degradation of NCoR1 by CMA**. (A, B) Primary hepatocytes were transfected with siLamp2a for 72 h or treated with AR7 10 μM for 24 h. Western blotting was used to quantify the protein levels of LAMP2A and CMA substrates. (C) Lysosome isolation was performed on siLamp2a-treated primary hepatocytes. Western blotting analysis was carried out to examine the protein expression of NCoR1 and LAMP2A in the lysosomal fraction. LAMP1 was used as a lysosomal marker and loading control. (D) Mouse primary hepatocytes, 72 h after transfection, were treated with cycloheximide 20 μM for the indicated times. Protein degradation of NCoR1 was determined by western blot analysis. The color code is black for siCon and red for siLamp2a. Each bar represents the mean ± SD (n = 3). Asterisks above the bars indicate significant differences compared to the control using Student's t-test: ∗p < 0.05; ∗∗p < 0.01.

To confirm that NCoR1 was degraded by LAMP2A-dependent lysosomal degradation, siLamp2a was introduced into primary hepatocytes to produce CMA-deficient cells. NCoR1 accumulated at the protein level, but not at the mRNA level, following treatment with siLamp2a (Figure 5A, Suppl 3A). LAMP2A expression levels increased and NCoR1 expression levels decreased in response to CMA activation by AR7 (Figure 5B, Suppl 3B). We further investigated whether NCoR1 translocates into lysosomes through LAMP2A. Lysosomal NCoR1 was not detected when cells were treated with siLamp2a (Figure 5C). In addition, western blot analysis of extracts from CMA-deficient mouse primary hepatocytes treated with cycloheximide confirmed that LAMP2A deficiency inhibited NCoR1 degradation (Figure 5D). These findings demonstrate that NCoR1 can be degraded by CMA via its interaction with Hsc70, implying that NCoR1 accumulation may play a role in PPARα suppression.

### 3.4 CMA inhibition inactivates PPARα and decreases fatty acid oxidation through NCoR1 degradation

**Figure 6:**
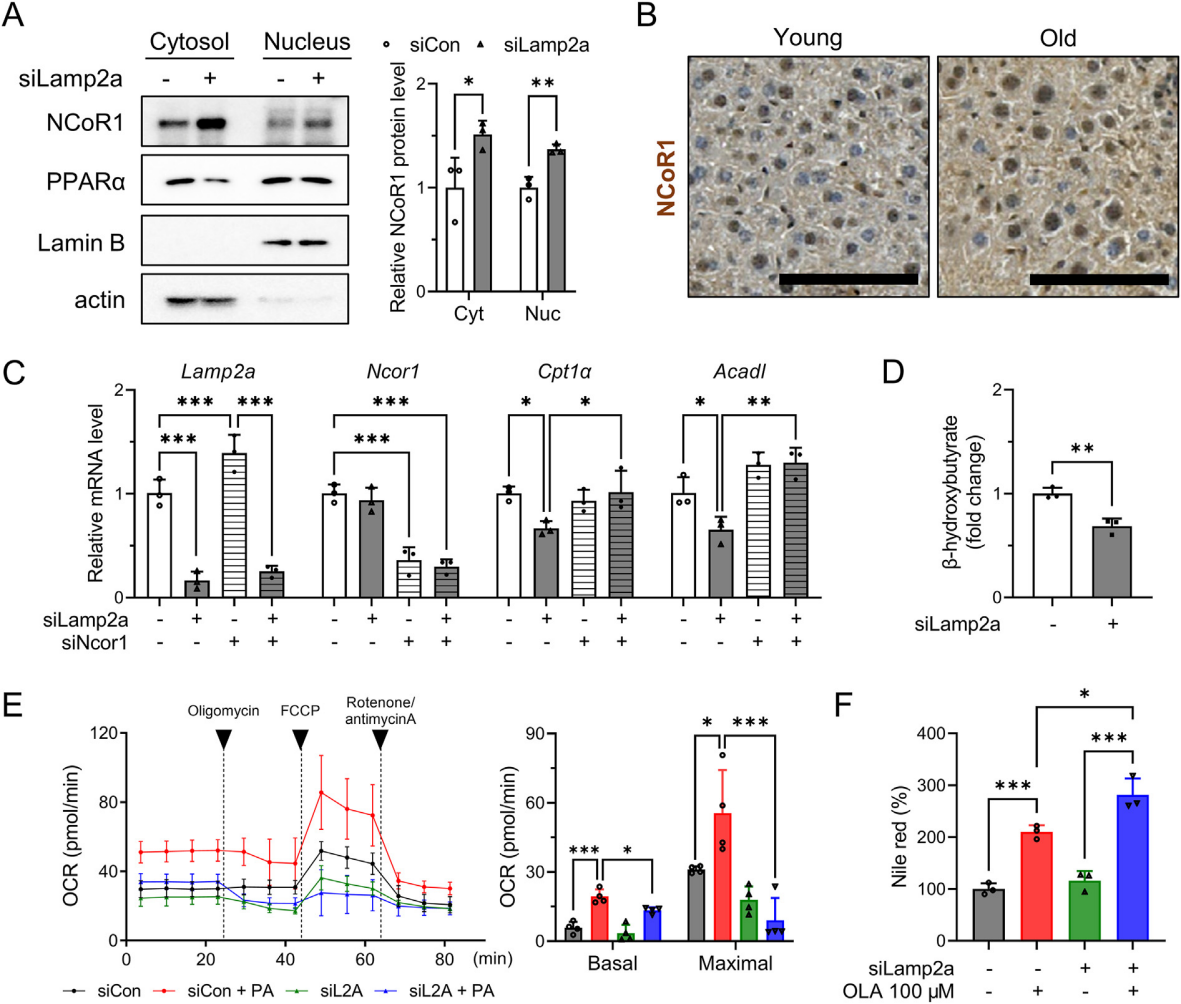
**Decreased fatty acid oxidation and increased lipid accumulation in CMA-deficient hepatocytes**. (A) Primary hepatocytes were transfected with siLamp2a for 72 h and subjected to nuclear fractionation. Cytosolic and nuclear protein expression of NCoR1 and PPARα were determined by western blotting. (B) Immunohistochemistry was performed to examine liver NCoR1 protein expression in young and old mice. Representative images are shown with a scale bar of 10 μm. (C) Primary hepatocytes were transfected with siLamp2a or siNcor1 for 72 h. cDNA was synthesized from isolated RNA and qRT-PCR was performed to examine the mRNA level of *Lamp2a*, *Ncor1*, *Cpt1α*, *Acadl*. (D) β-Hydroxybutyrate levels were measured in primary hepatocytes transfected with siCon or siLamp2a. (E) Palmitate oxidation rate of primary hepatocytes after siRNA transfection was measured by Seahorse analyzer. Cells were stimulated with BSA or palmitate-BSA following sequential treatment of oligomycin, FCCP, and rotenone/antimycin A. Basal and Maximal reparation were quantified ant presented in left panel. (F) Mouse primary hepatocytes were transfected with siLamp2a for 48 h and incubated with 200 μM oleic acid for 24 h. Nile red staining was used to measure the lipid content of cells. Each graph bar represents the mean ± SD (n = 3). Asterisks above the bars indicate significant differences compared to the control using Student's t-test when only two groups were compared. Multiple comparisons were performed using one-way ANOVA and Tukey's multiple comparisons test: ∗p < 0.05; ∗∗p < 0.01; ∗∗∗p < 0.001.

To determine whether the regulation of PPARα by CMA is NCoR1-dependent, lysates of CMA-deficient cells were subjected to nuclear fractionation. Inhibition of NCoR1 degradation in the cytoplasm of CMA-deficient cells led to a dramatic cytoplasmic and nuclear accumulation of NCoR1 (Figure 6A). Consistent with these results, the aged liver had higher protein levels of NCoR1 in the cytosol and nucleus (Figure 6B). Thus, CMA inhibition by siLamp2a or aging caused NCoR1 accumulation, leading to decreased expression of PPARα target genes (Figure 2A, B). To determine whether nuclear NCoR1 accumulation in CMA-deficient cells is associated with fatty acid oxidation, we initially investigated the regulatory effect of CMA inhibition on the expression of PPARα target genes through NCoR1. Remarkably, silencing Ncor1 restored the reduced levels of *Cpt1α* and *Acadl* mRNA in CMA-deficient cells (Figure 6C). Additionally, we evaluated the levels of β-hydroxybutyrate in primary hepatocytes treated with siCon or siLamp2a. CMA-deficient cells exhibited a significant reduction in β-hydroxybutyrate levels, indicating a decline in fatty acid oxidation (Figure 6D). Subsequently, we measured the OCR in control or CMA-deficient cells after BSA or palmitate-BSA treatment. When exposed to palmitate, CMA-deficient cells showed lower basal respiration and maximal respiration rates (Figure 6E). We further showed that CMA inhibition aggravated oleic acid-induced lipid accumulation, as measured using Nile red staining (Figure 6F).

NCoR1 has been reported to be selectively degraded by GABARAP-mediated macroautophagy [29]. As a decline in the levels of macroautophagy-related proteins has been observed during aging [30,31], we investigated whether macroautophagy also contributes to the accumulation of NCoR1. In our aging model, the hepatic expression of LC3 II/I, p62/SQSTM1, Atg5, and Atg7 was not affected by aging, suggesting that NCoR1 accumulation could occur independent of macroautophagy (Suppl 4). In our animal model, there was no change in the phosphorylation of AMPK and mammalian target of rapamycin (mTOR), an upstream regulator of macroautophagy (Suppl 5) [32]. These findings suggest that CMA inhibition, rather than macroautophagy, causes NCoR1 accumulation during aging.

**Figure 7:**
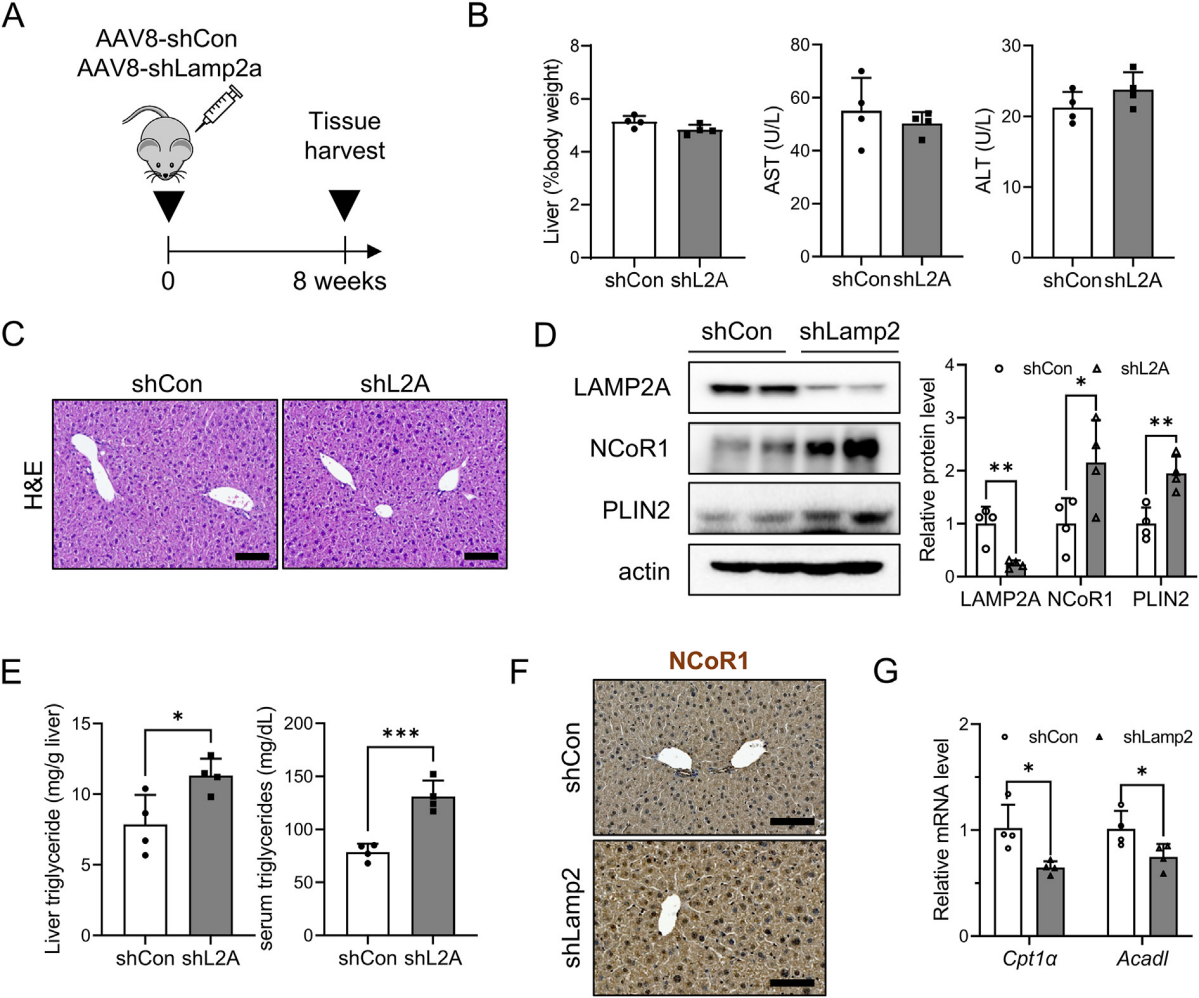
**NCoR1-dependent PPARα inactivation in CMA-deficient mouse liver**. (A) Experimental scheme illustrating AAV8-shRNA-mediated LAMP2A knockdown in mouse liver. (B) Body and liver weights were measured, and serum AST and ALT levels were determined as described in the Materials and methods section. (C) Representative images of H&E staining show the livers of CMA-deficient mouse liver, with a scale bar of 100 μm. (D) Hepatic protein levels of LAMP2A and CMA substrates were quantified using western blotting. (E) Triglyceride levels in liver and serum were quantified as described in the Materials and methods section. (F) Immunohistochemistry was performed to examine liver NCoR1 protein expression in CMA-deficient mice. Representative images are shown with a scale bar of 10 μm. (G) The hepatic mRNA levels of *Cpt1α* and *Acadl* were measured using qRT-PCR. Each graph bar represents the mean ± SD (n = 4). Asterisks above the bars indicate significant differences compared to the control using Student's t-test: ∗p < 0.05; ∗∗p < 0.01; ∗∗∗p < 0.001.

**Figure 8:**
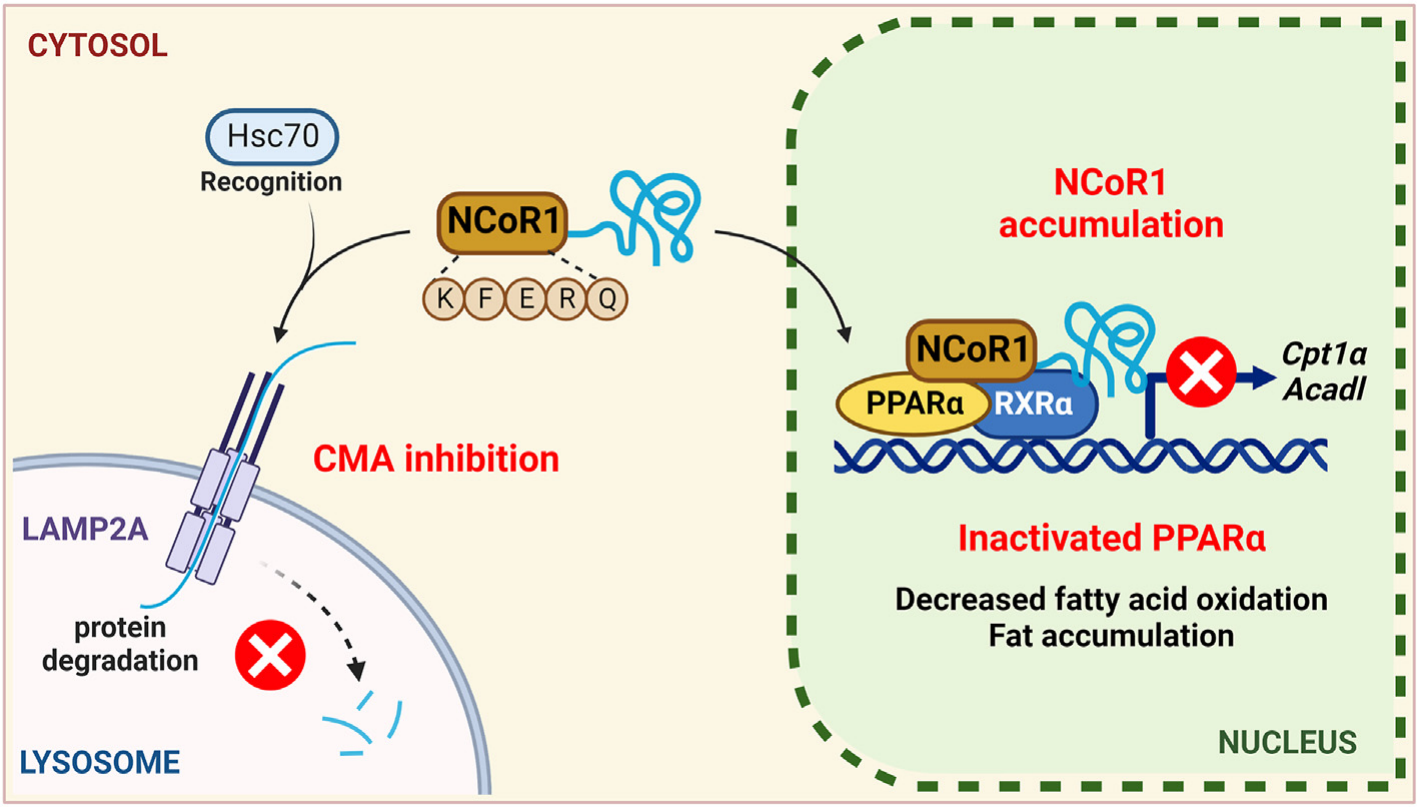
The underlying mechanisms of impaired fatty acid oxidation by CMA inhibition-mediated NCoR1 accumulation in the aged liver.

To examine the physiological relevance of NCoR1 and its accumulation in the context of CMA inhibition, 8-week-old male mice were injected with AAV8-shLamp2a intraperitoneally and euthanized after 8 weeks (Figure 7A). AAV8-shLamp2a injection efficiently silenced the hepatic protein levels of LAMP2A without causing liver toxicity (Figure 7B–D, Suppl 6). Western blot analysis also indicated that CMA inhibition in the liver induces the accumulation of PLIN2 (Figure 7D). Consistent with *in vitro* data in Figure 6F, liver triglyceride levels increased by 40% in CMA-deficient mice (Figure 7E). Serum triglyceride levels also increased in CMA-deficient mice. Histological analysis revealed greater nuclear NCoR1 levels in CMA-deficient mice than in control mice (Figure 7F). Consequently, the expression of fatty acid oxidation-related genes was significantly downregulated in CMA-deficient livers, while the expression of fatty acid uptake and de novo lipogenesis genes was unchanged. (Figure 7G, Suppl 7). As summarized in Figure 8, our data suggest that selective degradation of NCoR1 by CMA is decreased in the liver during aging, which is linked to decreased fatty acid oxidation and development of aging-related fatty liver.

## 4 Discussion

In this study, we described how CMA regulates hepatic lipid metabolism through NCoR1 degradation. Lipid accumulation during aging can be attributed to CMA-mediated NCoR1 accumulation and inhibition of fatty acid oxidation. We showed that NCoR1 contains a KFERQ-like motif and binds to Hsc70, which leads to lysosomal translocation and degradation. NCoR1 accumulation in CMA-deficient hepatocytes contributes to PPARα inactivation and decreases fatty acid oxidation. In contrast, pharmacological activation of CMA by AR7 facilitates NCoR1 degradation. Western blot analysis of aged livers also revealed decreased LAMP2A levels and increased NCoR1 levels, indicating that CMA is a key player in hepatic lipid metabolism. In liver-specific LAMP2A knockdown mice, we validated hepatic lipid accumulation, owing to a reduction in NCoR1 degradation.

NCoR1 was identified as a protein that interacts with nuclear receptors, such as PPARα [33]. The PPARα agonist WY-14643 reduces the interaction between NCoR1 and PPARα in a dose-dependent manner. In addition to the dissociation of NCoR1 in response to agonists, macroautophagy can modulate PPARα activity. Macroautophagy inhibition caused by ATG7 genetic ablation is accompanied by the accumulation of NCoR1 and defective fatty acid oxidation [29]. A number of recent studies have demonstrated that macroautophagy activity declines with age in rodents, highlighting its importance in hepatic function [15,[34], [35], [36], [37]]. However, in our aging model, we did not observe an age-associated decline in macroautophagy, as evidenced by the expression of autophagy-related proteins. Autophagy flux serves as a reliable indicator of macroautophagy activity, encompassing the dynamic macroautophagy process. It can be monitored by comparing the degradation of LC3 II and p62 in lysosomes, which involves measuring their levels in cells treated with and without lysosomal inhibitors. However, previous studies used LC3 II/I ratio and p62 accumulation as markers to monitor macroautophagy in the aged liver, leading to inconsistent results. For instance, Singh et al. demonstrated elevated levels of LC3 II/I and p62 in the aged liver, indicating late-stage autophagy inhibition [36], while Zhang and Cuervo reported an increase in LC3 II/I but no change in p62 level [15]. Furthermore, the analysis of the fractional volume of autophagic vacuoles in the aged liver suggests that macroautophagy may not significantly decrease with age [38]. These findings underscore the importance of considering technical limitations and inter-animal variability when measuring autophagic flux in mice, as macroautophagy may not always be inhibited simultaneously. Further investigation is required to assess autophagy flux in the aged liver using different techniques, considering their strengths and limitations. In this regard, employing autophagy-monitoring mouse models, such as transgenic mice expressing the GFP-LC3-RFP-LC3ΔG reporter, may offer a promising approach to gain deeper insights into macroautophagy activity during aging [39]. Nonetheless, our findings suggest that intact CMA activity, as well as macroautophagy, is required to maintain metabolic homeostasis in the aged liver.

To the best of our knowledge, this is the first study to demonstrate that CMA regulates fatty acid oxidation via NCoR1 degradation. Although previous studies have shown that CMA can degrade NCoR1 in various cancer cells, little is known about the mechanisms underlying CMA inhibition-mediated lipid accumulation [26,40]. Misfolded NCoR1 has been found in primary human cancer tissues derived from non-small cell lung cancer, and CMA-mediated degradation of misfolded NCoR1 promotes tumor cell survival by lowering ER stress [26]. NCoR1 degradation by CMA also influences the unfolded protein response and apoptosis in glioblastoma, indicating that CMA inhibition is also involved in cancer development [40]. Our findings demonstrate the potential involvement of CMA inhibition in aging-related fatty liver disease. CMA activity was reduced in aged livers, and NCoR1 accumulation caused by CMA inhibition was linked to impaired fatty acid oxidation. While previous studies have focused on the role of NCoR1 in tumor development, we demonstrated that NCoR1 may also play a role in aging-related fatty liver disease. NCoR1 can influence energy metabolism by repressing various nuclear receptors [41]. Defective autophagy results in NCoR1 accumulation, which inhibits liver X receptor (LXRα) transactivation and impairs de novo lipogenesis [42]. NCoR1 knockdown increases the amount of nuclear LXRα in macroautophagy-deficient cells and restores LXRα target gene expression involved in lipogenesis. Increased de novo lipogenesis in cancer cells may be a response to the high metabolic demand for structural components and signaling molecules, which is linked to tumor progression [43]. According to a recent study, hepatocyte-specific NCoR1 deficiency promotes de novo lipogenesis and enhances liver regeneration after partial hepatectomy [44]. NCoR1 deficiency, on the other hand, fails to suppress de novo lipogenesis in diethylnitrosamine-treated mice and worsens carcinogenic liver injury. Since lipid metabolism is an integral component of the pathogenesis of cancer, further investigation is needed to determine the relevance of CMA-mediated metabolic changes in tumor development.

CMA inhibition during aging regulates hepatic lipid metabolism and has several pathophysiological implications. Aged mouse liver exhibits decreased LAMP2A expression and hepatic steatosis [15,16]. A liver-specific LAMP2A knockout affects glucose and lipid homeostasis [11]. CMA degrades the enzymes involved in glucose and lipid metabolism, and CMA deficiency contributes to metabolic abnormalities. Moreover, the livers of aged CMA-deficient mice are susceptible to oxidative stress, resulting in accelerated hepatic dysfunction that manifests as decreased drug metabolism and metabolic dysregulation [16]. In our aging model, LAMP2A downregulation and the subsequent CMA inhibition were accompanied by hepatic lipid accumulation. The decreased expression of genes involved in fatty acid oxidation underlies the altered lipid metabolism observed in the CMA-deficient hepatocytes. Downregulation of LAMP2A expression in hepatocytes or mouse liver results in PPARα inactivation via NCoR1 accumulation, decreased PPARα target genes, and decreased fatty acid oxidation. There is a possibility that the repression of *Cpt1a* and *Acadl* in CMA-deficient hepatocytes is driven by a mechanism independent of PPARα inactivation. For example, it has been demonstrated that PGC1α binding to CCAAT/enhancer binding protein β (CEBPβ) can induce the upregulation of *Cpt1a* gene in nasopharyngeal carcinoma cells [45]. Since we have not demonstrated the direct recruitment of PPARα or NCoR1 to response elements in the promoters of these genes, further investigation is needed to validate the repression of *Cpt1a* and *Acadl* gene expression through PPARα interaction.

Aging-induced CMA inhibition leads to hepatic lipid accumulation by impairing the degradation of PLIN2, a protein associated with lipid metabolism and a substrate of CMA. When LAMP2A is knocked out, CMA activity is reduced, leading to elevated levels of PLIN2 expression, decreased lipid oxidation, and the subsequent accumulation of lipid droplets [8]. These significant changes in the two lipid metabolic processes may be the underlying mechanism responsible for the lipid accumulation in the aged liver. Despite the age-related increase in SREBP1 and lipogenic gene expression in mice, suppressing LAMP2A in mouse hepatocytes or in the liver of mice does not affect their expression. These findings strongly indicate that the reduction in CMA activity and the increase in NCoR1 do not contribute to the activation of lipogenic genes observed in aged mice. It can be inferred that an alternative mechanism, unrelated to CMA, is responsible for the induction of lipogenic genes in old mice. Therefore, our results suggest that CMA inhibition, which regulates lipid catabolism, may be important for the development of aging-related fatty liver disease. Genetic manipulation to maintain CMA activity in aged livers has been shown to improve metabolic abnormalities in aged mice [15]. CMA can be investigated as a therapeutic target to manage aging-related fatty liver because fatty acid catabolism in the liver is important during times of increased lipid burden.

In conclusion, we determined CMA activity in the aged liver and discovered that CMA inhibition and NCoR1-mediated PPARα inactivation are responsible for aging-related fatty liver. NCoR1 is translocated into the lysosome after Hsc70 recognizes the KFERQ-like motif. CMA-mediated NCoR1 degradation decreases with age, and NCoR1 accumulates in the nucleus, leading to PPARα inactivation. LAMP2A knockdown in the mouse liver resulted in hepatic steatosis, demonstrating that the NCoR1/PPARα pathway inhibited fatty acid oxidation. Owing to the association between metabolic homeostasis and aging-related disorders, we speculate CMA to be a potential therapeutic target for controlling hepatic lipid metabolism during aging.

## Animal welfare and ethics statement

Ethical approval for this study was obtained from the Institutional Animal Care and Use Committee of Seoul National University.

## Funding

This study was supported by the Basic Science Research Program through the 10.13039/501100003725National Research Foundation of Korea (NRF), funded by the Ministry of Education [NRF-2021R1C1C2004529, NRF-2022R1A6A1A03046247, NRF-2023R1A2C2004929].

## Author contributions

**You-Jin Choi**: Conceptualization, Methodology, Validation, Formal analysis, Investigation, Writing – original draft, Writing – review & editing, Visualization, Funding acquisition. **Sung Ho Yun**: Methodology, Validation, Formal analysis, Investigation. **Jihyeon Yu**: Methodology, Investigation, Resources. **Yewon Mun**: Validation, Investigation. **Wonseok Lee**: Validation, Investigation. **Cheon Jun Park**: Methodology, Investigation, Writing – original draft. **Byung Woo Han**: Methodology, Writing – original draft, Writing – review & editing. **Byung-Hoon Lee**: Conceptualization, Writing – original draft, Writing – review & editing, Supervision, Project administration, Funding acquisition.

## Declaration of competing interest

The authors have no conflicts of interest to disclose.

## Data Availability

Data will be made available on request.
