# Supplementary material for: Chaperone-mediated autophagy dysregulation during aging impairs hepatic fatty acid oxidation via accumulation of NCoR1
Source: Mol Metab. 2023 Jul 29;76:101784. doi: 10.1016/j.molmet.2023.101784 (PMC10448198; doi:10.1016/j.molmet.2023.101784)
Supplement: Multimedia component 1 [file mmc1.docx]

**Chaperone-mediated autophagy dysregulation during aging** **impairs hepatic fatty acid oxidation via accumulation of NCoR1**

You-Jin Choi^1^, Sung Ho Yun^1^, Jihyeon Yu^2^, Yewon Mun^1^, Wonseok Lee^1^, Cheon Jun Park^1^, Byung Woo Han^1^, and Byung-Hoon Lee^1,*^

^1^ College of Pharmacy and Research Institute of Pharmaceutical Sciences, Seoul National University, Seoul 08826, Republic of Korea

^2^ Division of Life Science, Korea Polar Research Institute, Incheon 21990, Republic of Korea

* Correspondence: Byung-Hoon Lee

Address: 1, Gwanak-ro, Gwanak-gu, Seoul, Republic of Korea, 08826

E-mail: lee@snu.ac.kr

Tel: 82-2-880-7843

**Supplementary Methods**

***Microarray data analysis***

Microarray datasets deposited in NCBI Gene Expression Omnibus (GEO) under accession number GSE3150 were retrieved for analysis [1]. The datasets included samples from male wild type mice aged 4 and 22 months (n=5). Pre-processed data available in GEO were analyzed using GEO integrated analyzing tool (GEO2R) and GraphPad Prism7.0 software. Statistical comparisons were done using Student’s t-test and probability values were corrected for multiple testing with the Benjamini–Hochberg multiple-testing correction for FDR control of the result.

**References**

[1] Boylston, W.H., DeFord, J.H., Papaconstantinou, J., 2006. Identification of longevity-associated genes in long-lived Snell and Ames dwarf mice. Age (Dordr) 28(2):125-144.

**Supplementary data**

**
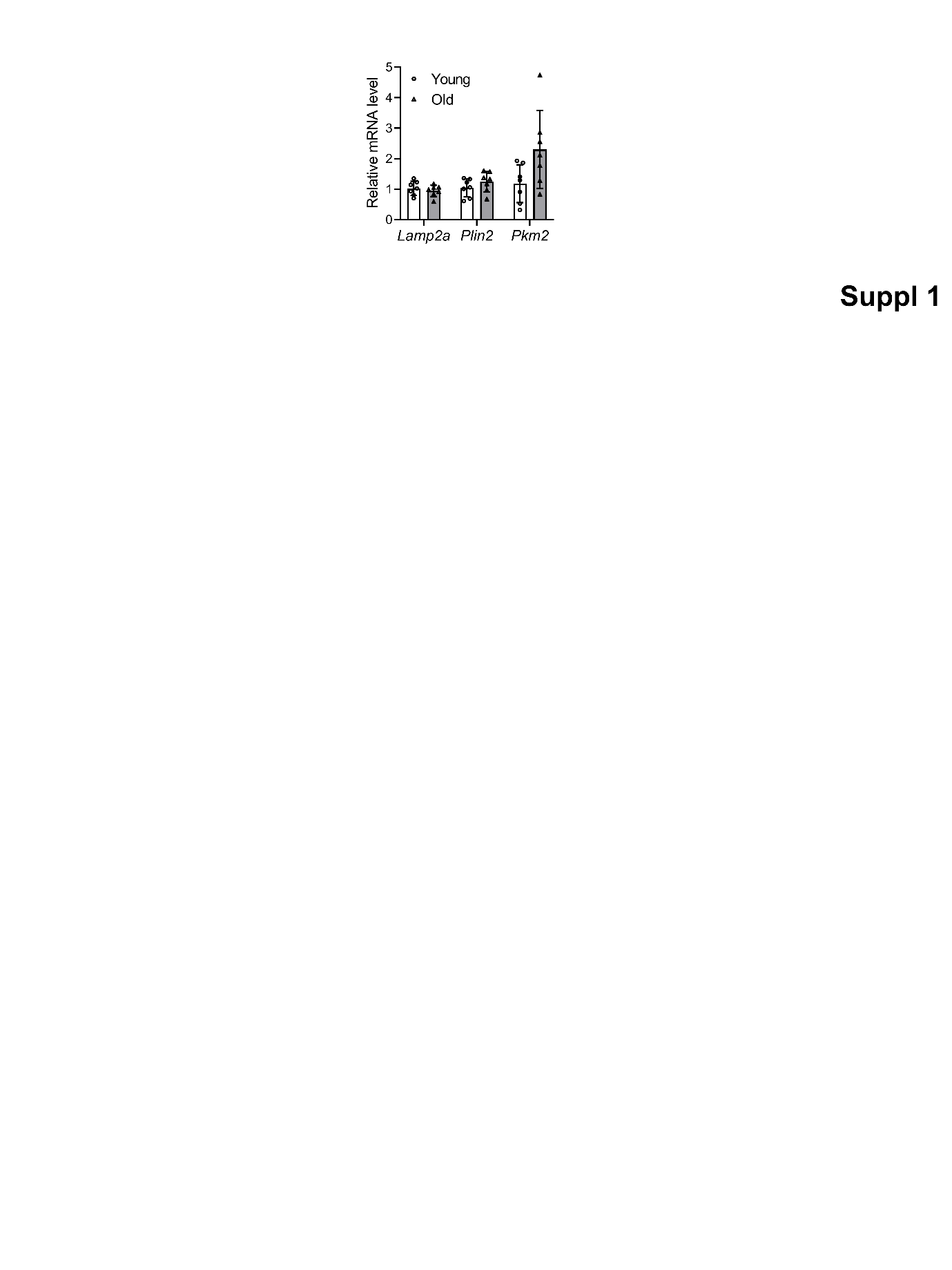
**

**Supplementary Fig 1. Analysis of mRNA levels of *Lamp2a* and CMA substrates in livers from mice of different ages**

Livers from mice of two different age groups (3 and 22 months) were used for RNA extraction. qRT-PCR was performed to measure hepatic mRNA levels of *Lamp2a* and CMA substrates (*Plin2* and *Pkm2*). Each bar represents the mean ± SD (n=7). Comparison between two groups was performed using Student's t-test.

| Gene symbol | Fold change | p value | adj p value |
| --- | --- | --- | --- |
| *Cpt1α* | 0.819 ± 0.132 | 0.192 | 0.507 |
| *Cpt2* | 0.710 ± 0.057 | 0.016 | 0.195 |
| *Acadl* | 0.748 ± 0.025 | 0.014 | 0.190 |
| *Acox1* | 0.755 ± 0.129 | 0.063 | 0.318 |
| *Srebf1* | 0.620 ± 0.250 | 0.028 | 0.236 |
| *Acaca* | 2.047 ± 0.352 | 0.019 | 0.207 |
| *Fasn* | 0.775 ± 0.417 | 0.341 | 0.642 |
| *Cd36* | 0.579 ± 0.179 | 0.065 | 0.323 |
| *Dgat2* | 0.852 ± 0.136 | 0.184 | 0.499 |
| *Mttp* | 0.803 ± 0.131 | 0.087 | 0.363 |


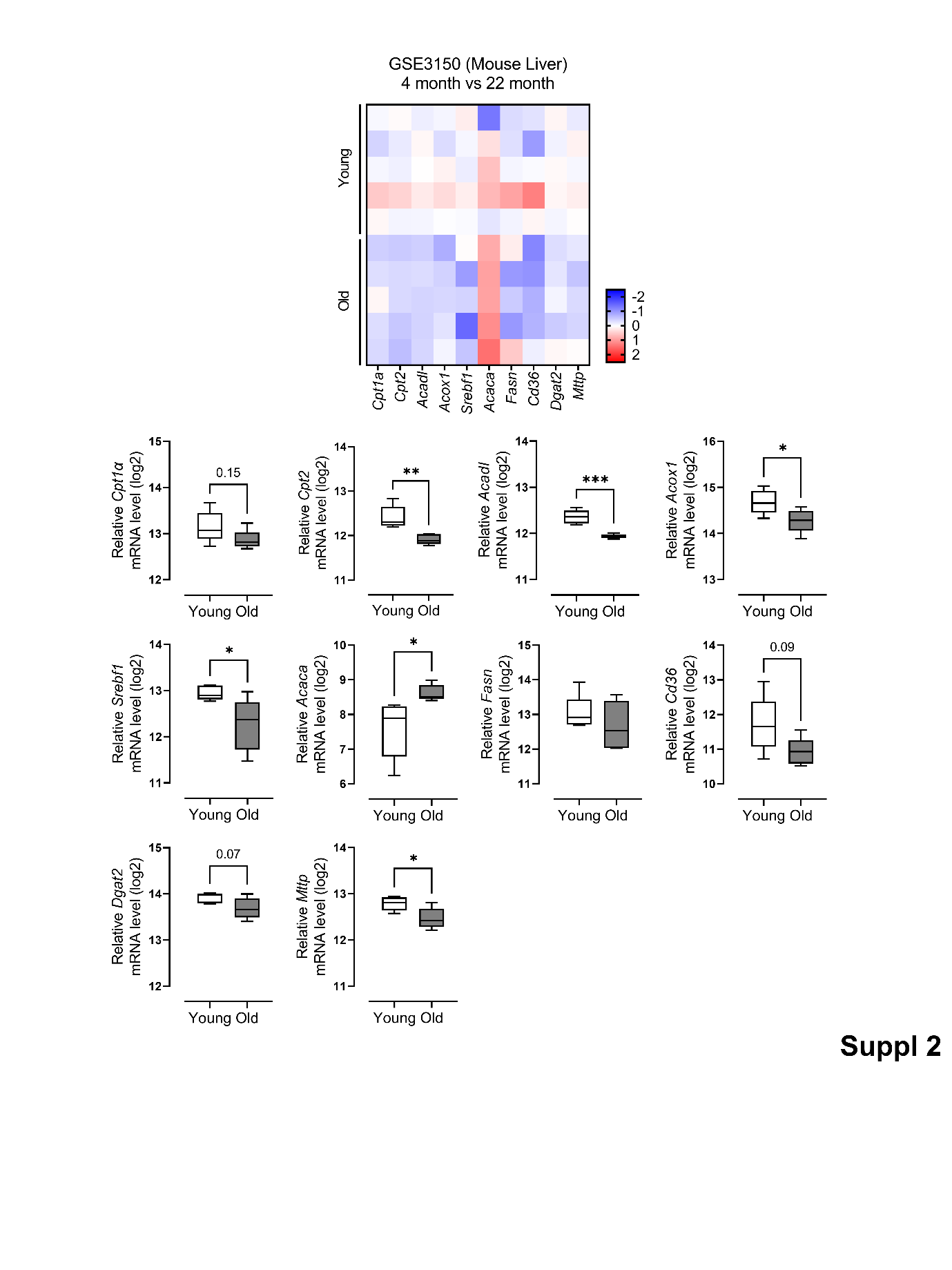


**Supplementary Fig 2. Profiling of lipid metabolism-related genes in the mouse liver at different ages using microarray data from GSE3150**

Livers from mice of two different age groups (4 and 22 months) were used for microarray analysis (GSE3150). The left panel shows the heatmap of genes associated with lipid metabolism, with each column representing an individual gene involved in the pathway. The right panel displays the fold change of mRNA levels for lipid metabolism-related genes. The microarray data were analyzed by Student’s t-tests, and the values presented are mean ± SD (n=5). The p-values of these tests were adjusted for multiple testing using the Benjamini-Hochberg false discovery rate approach.

**
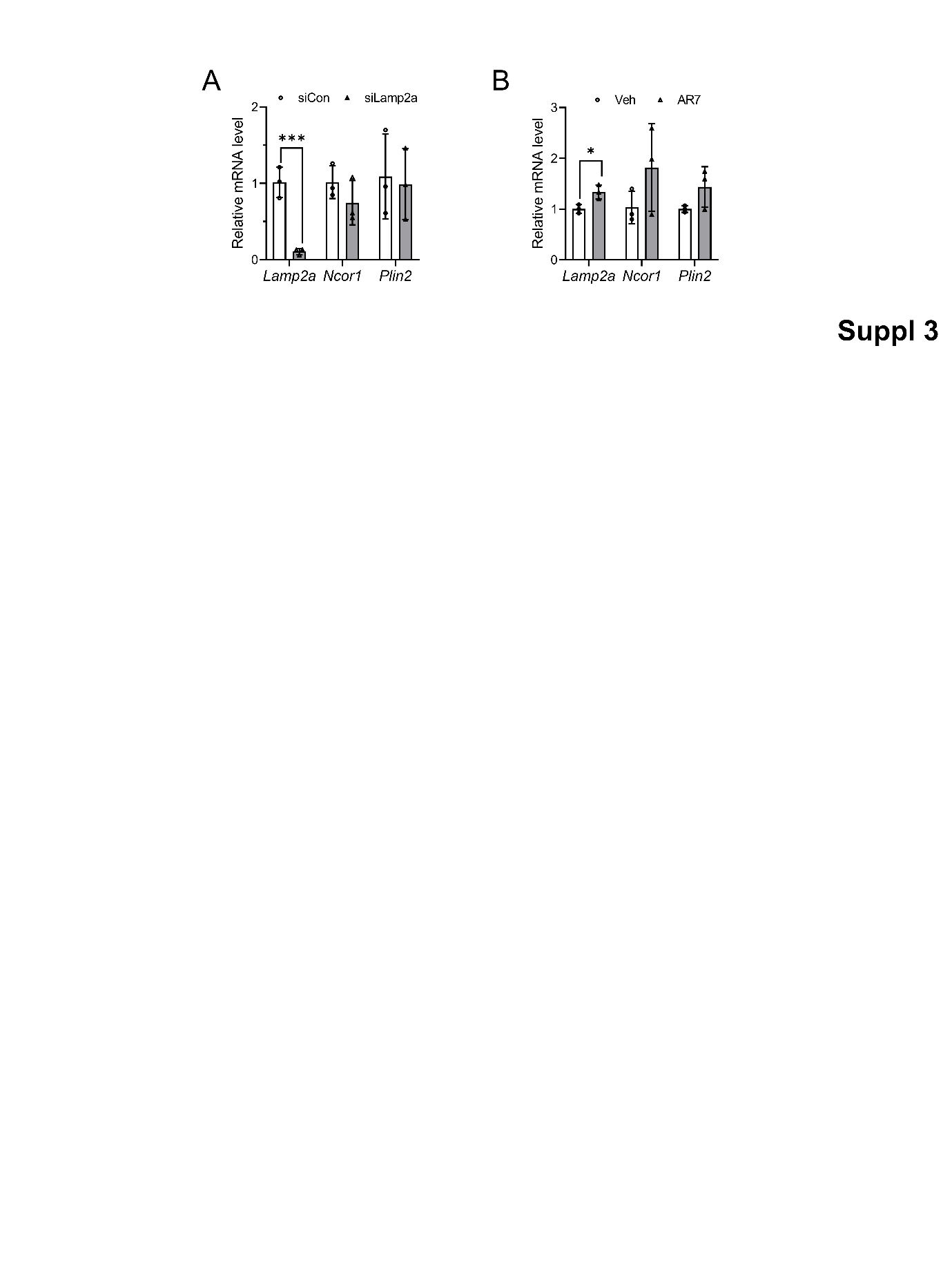
**

**Supplementary Fig 3. Analysis of mRNA levels of *Lamp2a, Ncor1, and Plin2* in CMA-deficient hepatocytes or AR7-treated hepatocytes**

Primary hepatocytes were transfected with siLamp2a for 72 h or treated with AR7 10 μM for 24 h. cDNA was synthesized from isolated RNA and qRT-PCR was performed to examine the mRNA level of *Lamp2a, Ncor1,* and *Plin2*. Each graph bar represents the mean ± SD (n=3). Asterisks above the bars indicate significant differences compared to the control using Student's t-test: *, p<0.05; ***, p<0.001.


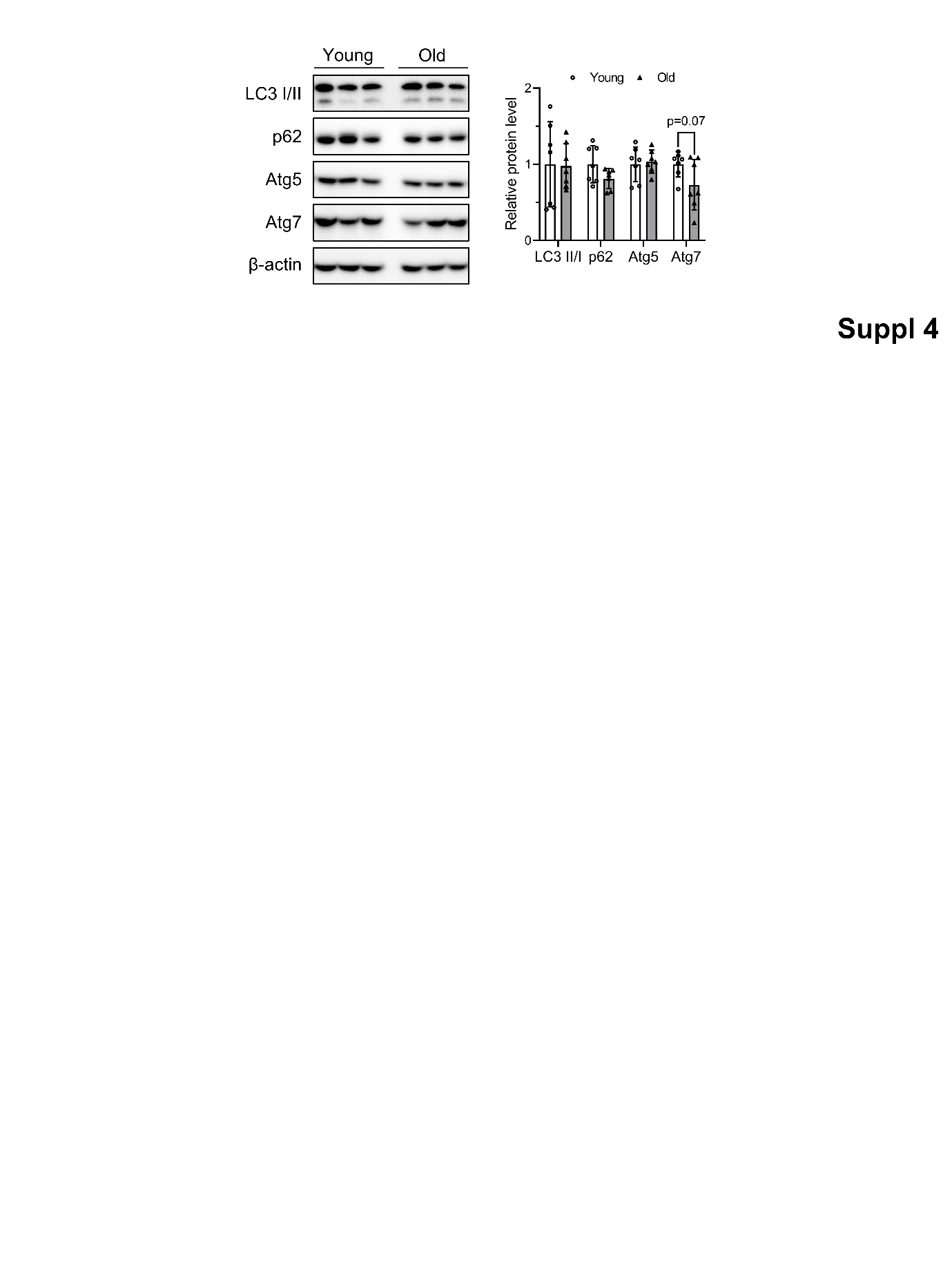


**Supplementary Fig 4. Analysis of expression levels of macroautophagy-related proteins in livers from mice of different ages**

Livers from mice of two different age groups (3 and 22 months) were used for protein extraction. Hepatic expression levels of autophagy-related proteins were quantified using western blotting. Each graph bar represents the mean ± SD (n=7). Comparison between two groups was performed using Student's t-test.

**
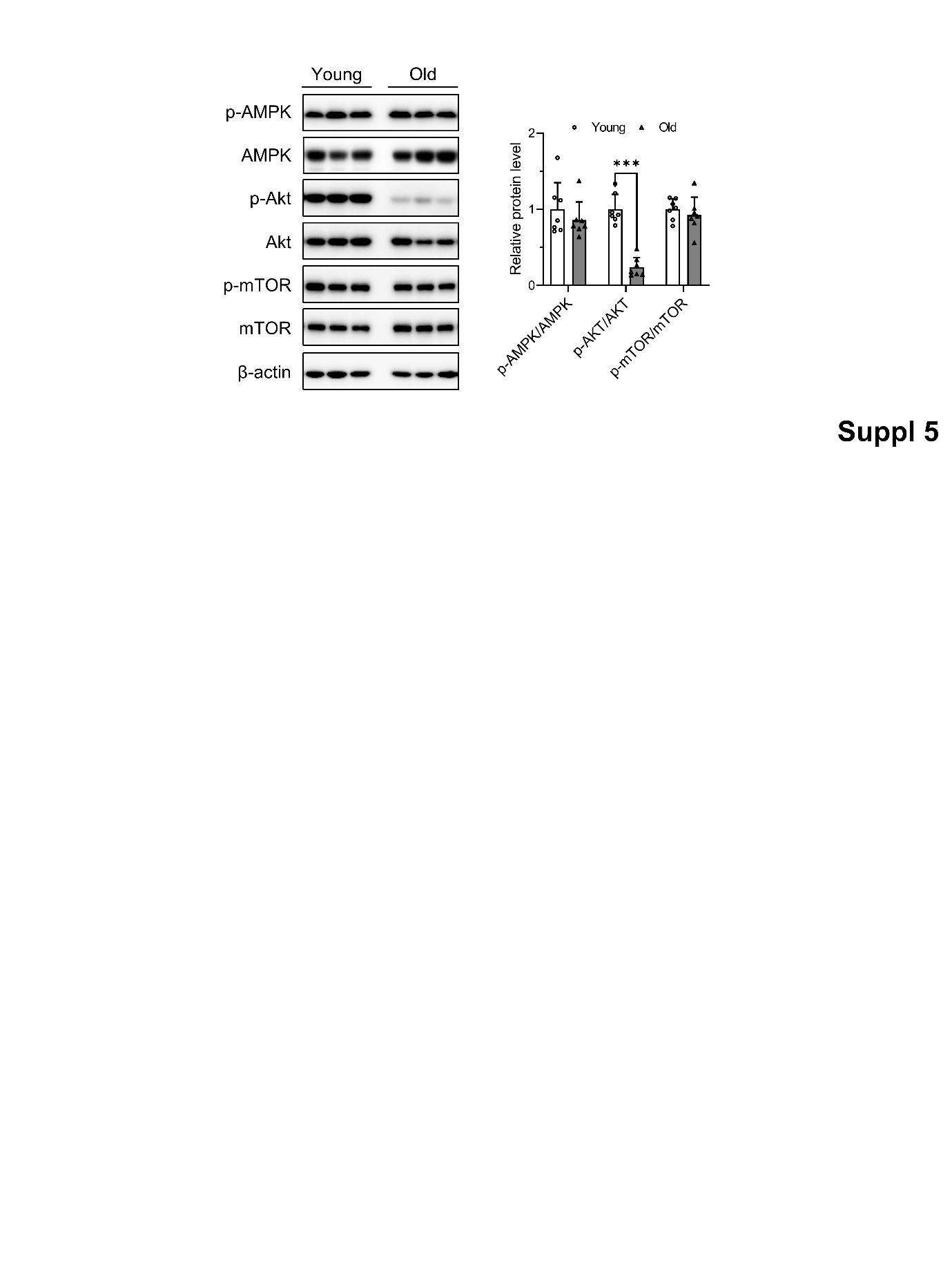
**

**Supplementary Fig 5. Analysis of protein expression levels of upstream regulator of macroautophagy in livers from mice of different ages**

Livers from mice of two different age groups (3 and 22 months) were used for protein extraction. Hepatic expression levels of upstream regulator of macroautophagy were quantified using western blotting. Each graph bar represents the mean ± SD (n=7). Asterisks above the bars indicate significant differences compared to young mice using Student's t-test: ***, p<0.001.

**
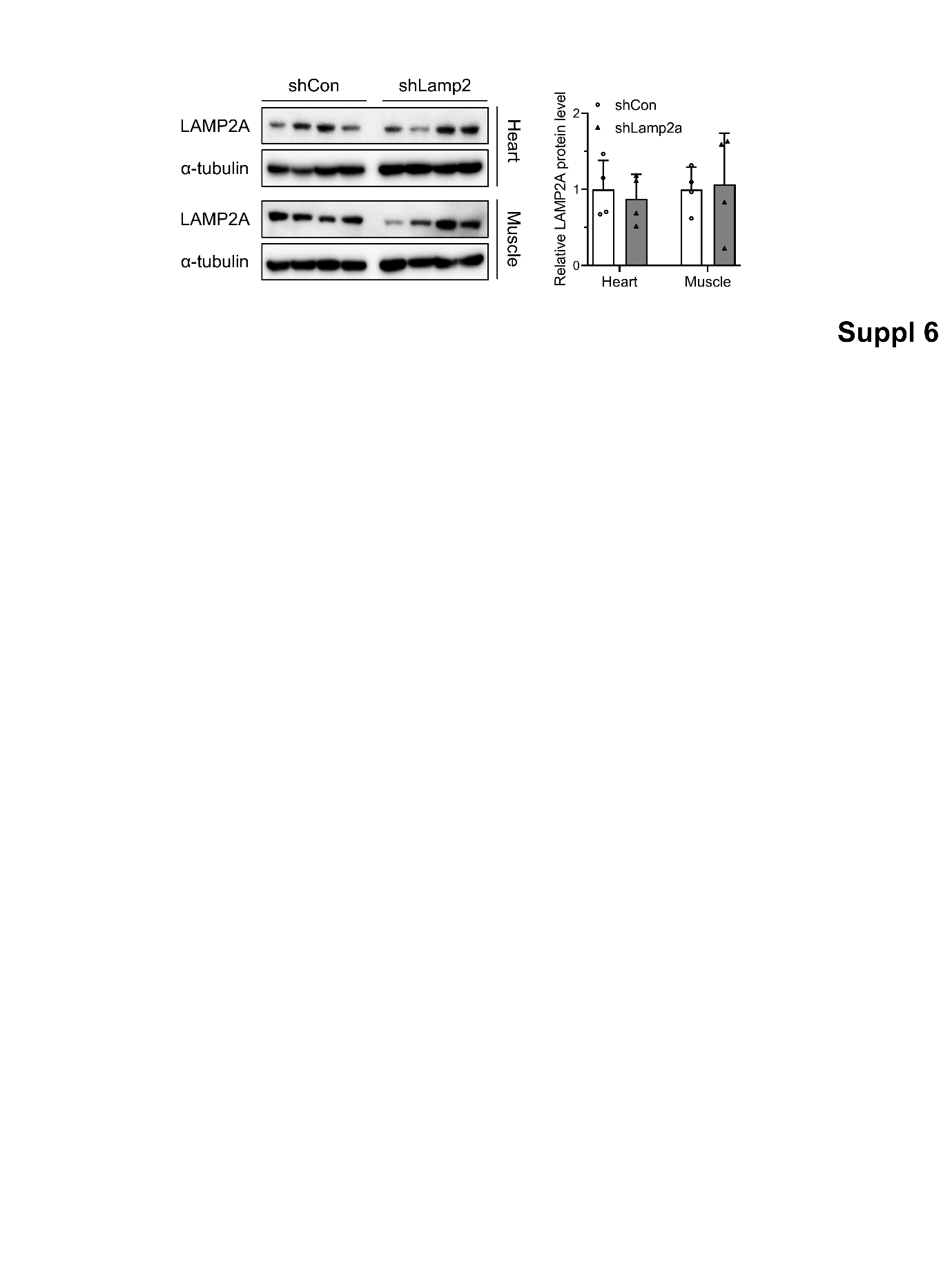
**

**Supplementary Fig 6. Liver-specific knockdown of LAMP2A to generate CMA-deficient mouse**

Mice were injected with AAV8-shLamp2a intraperitoneally and euthanized after 8 weeks. Protein levels of LAMP2A in heart and muscle were quantified using western blotting. Each graph bar represents the mean ± SD (n=4). Comparison between two groups was performed using Student's t-test.


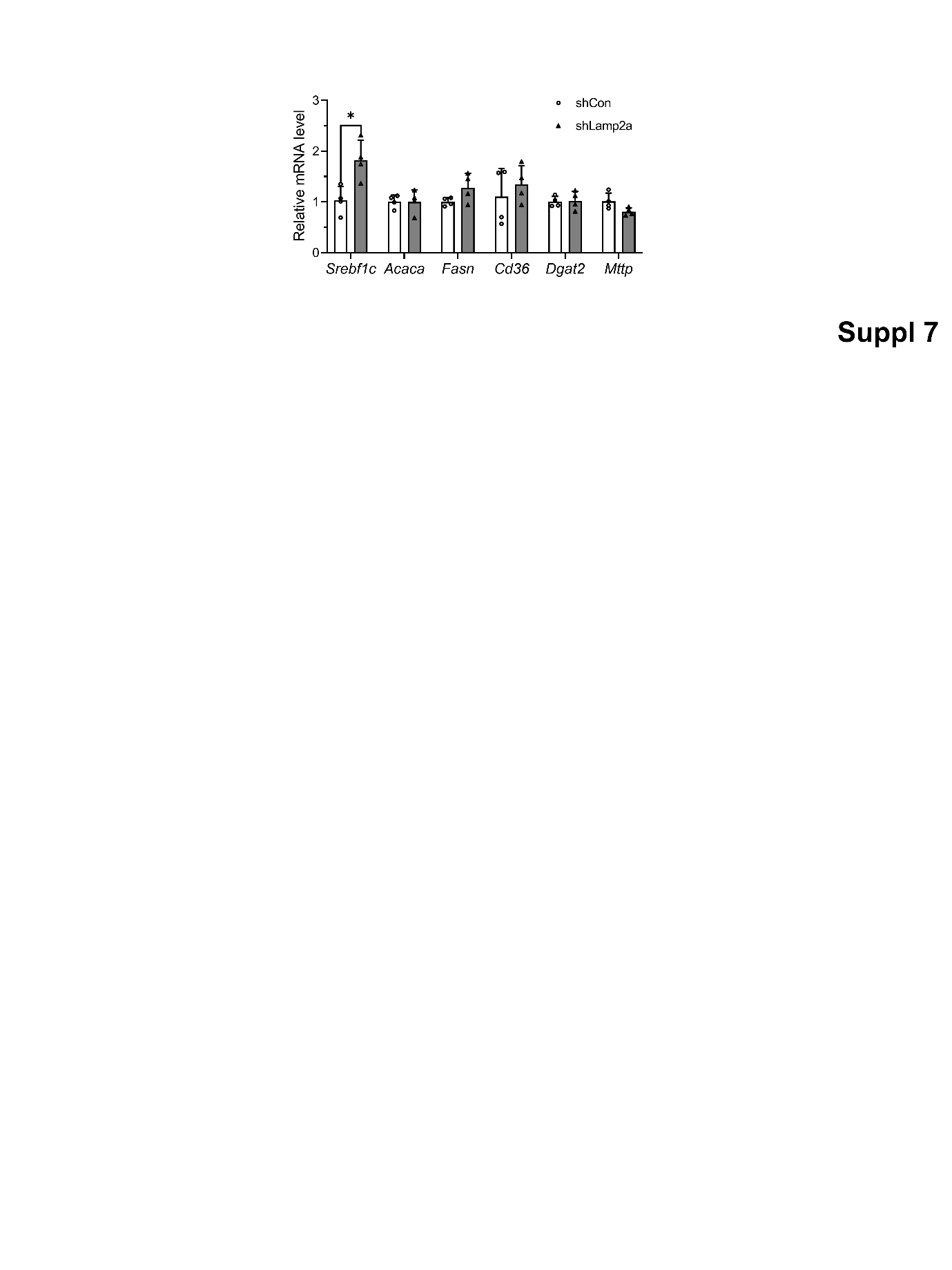


**Supplementary Fig 7. Analysis of mRNA levels of fatty acid uptake and *de novo* lipogenesis genes in CMA-deficient hepatocytes**

Primary hepatocytes were transfected with siCon or siLamp2a for 72 h. cDNA was synthesized from isolated RNA and qRT-PCR was performed to examine the mRNA level of fatty acid uptake and *de novo* lipogenesis genes. Each graph bar represents the mean ± SD (n=3). Asterisks above the bars indicate significant differences compared to the control using Student's t-test: *, p<0.05.
